# Impact of dehydration on the physiochemical properties of *Nostoc calcicola* BOT1 and its untargeted metabolic profiling through UHPLC-HRMS

**DOI:** 10.3389/fpls.2023.1147390

**Published:** 2023-06-23

**Authors:** Priya Yadav, Rahul Prasad Singh, Hissah Abdulrahman Alodaini, Ashraf Atef Hatamleh, Gustavo Santoyo, Ajay Kumar, Rajan Kumar Gupta

**Affiliations:** ^1^ Laboratory of Algal Research, Centre of Advanced Study in Botany, Institute of Science, Banaras Hindu University, Varanasi, India; ^2^ Department of Botany and Microbiology, College of Science, King Saud University, Riyadh, Saudi Arabia; ^3^ Instituto de Investigaciones Químico-Biológicas, Universidad Michoacana de San Nicolás de Hidalgo, Morelia, Mexico

**Keywords:** *Nostoc calcicola*, dehydration, chlorophyll fluorescence, osmoprotectants, metabolomics, UHPLC-HRMS

## Abstract

The global population growth has led to a higher demand for food production, necessitating improvements in agricultural productivity. However, abiotic and biotic stresses pose significant challenges, reducing crop yields and impacting economic and social welfare. Drought, in particular, severely constrains agriculture, resulting in unproductive soil, reduced farmland, and jeopardized food security. Recently, the role of cyanobacteria from soil biocrusts in rehabilitating degraded land has gained attention due to their ability to enhance soil fertility and prevent erosion. The present study focused on *Nostoc calcicola* BOT1, an aquatic, diazotrophic cyanobacterial strain collected from an agricultural field at Banaras Hindu University, Varanasi, India. The aim was to investigate the effects of different dehydration treatments, specifically air drying (AD) and desiccator drying (DD) at various time intervals, on the physicochemical properties of *N. calcicola* BOT1. The impact of dehydration was assessed by analyzing the photosynthetic efficiency, pigments, biomolecules (carbohydrates, lipids, proteins, osmoprotectants), stress biomarkers, and non-enzymatic antioxidants. Furthermore, an analysis of the metabolic profiles of 96-hour DD and control mats was conducted using UHPLC-HRMS. Notably, there was a significant decrease in amino acid levels, while phenolic content, fatty acids, and lipids increased. These changes in metabolic activity during dehydration highlighted the presence of metabolite pools that contribute to the physiological and biochemical adjustments of *N. calcicola* BOT1, mitigating the impact of dehydration to some extent. Overall, present study demonstrated the accumulation of biochemical and non-enzymatic antioxidants in dehydrated mats, which could be utilized to stabilize unfavorable environmental conditions. Additionally, the strain *N. calcicola* BOT1 holds promise as a biofertilizer for semi-arid regions.

## Introduction

The rising concentrations of CO_2_ in the atmosphere are causing significant changes in meteorological patterns and reductions in precipitation in the majority of the globe. In these alarming scenarios, cyanobacteria seem to be an efficient and potential candidate for the protection of soil erosion and detention (Wigley and Jones, 1985; Belnap et al., 2016; Ebi and Loladze, 2019; WMO, 2021). Exopolysaccharide (EPS) produced by cyanobacteria aids in nutrient management and water retention in the medium, producing favorable circumstances for microbe survival in nutrient-limited and dry environments (Colica et al., 2014; Chamizo et al., 2019).

In previous studies, various authors have reported on drought-tolerant cyanobacteria and their use as biofertilizers for non-water-logging crops (Abd-Alla et al., 1994; Kuraganti et al., 2020). In addition, the bio-priming of seeds like *Acacia hilliana*, *Senna notabilis, Grevillea wickhamii*, *Eucalyptus gamophylla*, and *Oryza sativa* with *Microcoleus* sp., *Anabaena oryzae*, *Nostoc punctiformae*, and *Nostoc* sp. resulted in improved the germination and growth of seeds under drought conditions (Chua et al., 2020; Yadav et al., 2022a).

In biocrust, some cyanobacteria have been reported as dominant and pioneer organisms, which promote the succession of pioneer species *via* secreting EPS and increase soil fertility (Lan et al., 2013; Park et al., 2017; Mugnai et al., 2018). Some of the cyanobacterial species like *Calothrix parietina, Scytonema crispum, Scytonema hyalinum, Nostoc* sp., *N. calcicola*, and *N. commune* commonly form biocrusts on dry lands, resulting in survivability in extreme desiccation and heat environments (Dojani et al., 2014; Büdel et al., 2016; Becerra-Absalón et al., 2019; Roncero-Ramos et al., 2019).

Several cyanobacteria have the ability to grow and thrive even in extremely dry environments, and these cyanobacterial species play a crucial role in the maintenance of moisture content, solubilization and mobilization of phosphorus and nitrogen fixation, etc. (Deinlein et al., 2014; Garlapati et al., 2019; Gr et al., 2021). Therefore, these cyanobacterial strains can be used for draught stress management. However, relatively little evidence has been reported on the cyanobacterial-mediated improvement of plant growth under harsh environmental conditions, like drought, low and high temperatures, ultraviolet radiation, freezing, and hot springs (Castenholz, 1992; Zakhia et al., 2008; Tashyreva and Elster, 2016; Yadav et al., 2022b). *N. calcicola* is an alkaliphilic halotolerant, filamentous nitrogen-fixing cyanobacterium, generally grown in the range of 0.5 to 2 M concentrations of salt (Singh V, et al., 2015). In previous studies, cyanobacterial species like *N. calcicola*, *Nostoc commune*, and *Microcoleus* sp. have been effectively used in the restoration of sodic lands and saline areas because they are remarkably tolerant to salt stress (Thapar et al., 2008). Studies have reported that *Anabaena variabilis* and *N. calcicola* can withstand NaCl concentration in the range of 0.1 to 0.8 M and effectively sequester heavy metals such as Cd(II), Cu(II), and Co(II) (Usmonkulova et al., 2022). *A. variabilis* and *N. calcicola* have also been found to reduce the chlorogenic compound hexachlorocyclohexane (Kadirova et al., 2012a). *N. calcicola* not only acts as a biofertilizer but also as a biostimulator of the development and growth of higher plants by synthesizing the growth hormones auxin and gibberellins (Kadirova and Shakirov, 2012b; Yadav et al., 2022a).

In previous studies, various authors have reported the growth of cyanobacteria strains on granite, sandstone, limestone, and marble (Singh V, et al., 2015). However, under extreme conditions, they form biofilms composed of EPSs containing polysaccharides, glycoproteins, lipopolysaccharides, glycolipids, and other extracellular enzymes (Yang et al., 2009). The EPS produced by these cyanobacterial strains helps in its protection and crust formation.

Recent developments in “omics” technologies have made it possible to compare the amounts of chemical compounds in desiccation-tolerant versus desiccation-sensitive organisms by quantitatively monitoring their abundance in a high-throughput way. The Ultra-high-performance liquid chromatography high-resolution mass spectrometry (UHPLC-HRMS) method can identify metabolites in low concentrations and has a higher capacity to limit false discovery rates (Khan et al., 2019).

In this present study, we evaluated the effect of desiccation on various biochemical properties like pigments, proteins, carbohydrates, and lipids of *N. calcicola* BOT1. We also evaluated osmoprotectants, stress biomarkers, and non-enzymatic antioxidant tests to understand the survival mechanism of the strain under dehydration stress. Furthermore, we performed UHPLC-HRMS to detect metabolites from the control and 96 h DD mats to understand how these metabolites relate to the organism’s survivability in drought conditions.

## Material and methods

### Isolation and purification of the cyanobacterial strain

The filamentous test organisms were collected from the rice field located at the B.H.U. Varanasi, India campus. Then, the samples were serially diluted and cultured in the basal growth medium (BG-11N^-^) nitrogen-free broth medium for 15 days before being spread on the agar plates (Rippka et al., 1979).

The cyanobacterial colonies grown on the agar plate were picked aseptically and transferred to 100 ml BG-11N^-^ containing medium in 250 ml flasks. Then, further isolated colonies were transferred to a new BG-11N^-^ plate and incubated at 25 ± 2°C, under the 55 μmol photons m^-2^s^-1^ illumination provided by a cool fluorescent tube light.

### Culture conditions

The mother cultures were grown in a conical flask with 200 ml of BG-11N^-^ (pH 7.4) medium for 25 days at 25 ± 2°C under 55 μmol photons m^-2^s^-1^ light intensity with a 14:10 h day-night photoperiod. The growth media containing cyanobacteria were manually shaken 3–5 times a day for proper growth and suspension formation. Then, 10% mother cultures of isolates (at exponential phase) were aseptically transferred into a 2 L conical flask containing 1300 ml of BG-11 N^-^ medium and grown under the above-described culture conditions.

### Identification of isolate by light and scanning electron microscopy

The isolates were first observed under light microscopy by preparing temporary slides. The shape, size, and color of the thallus, the width and length of the trichome, the presence and position of heterocysts, branching in filaments, hormogonia, and akinetes were considered during taxonomic characterization (Desikachary, 1959; Komárek, 2013). The detailed morphology of the isolates was visualized using SEM. Cyanobacterial filaments were placed at the center of the cover slip, dried, and chemically fixed with 2.5% (v/v) glutaraldehyde. After complete drying, they were washed with double-distilled water (DDW) then further dehydrated with increasing ethanol concentrations (30%, 50%, 90%, and 100%) and again air dried. An Sc 7620 sputter coater was used to coat the dried cyanobacterial samples with gold-palladium at a thickness of 30Å for 5 min. Further, these coated samples were used for their morphological identification under SEM (EVO18 Research ZEISS- Germany) (Sadiq et al., 2011).

### Molecular characterization of cyanobacteria isolates

For molecular characterization genomic DNA of the cyanobacteria isolate was extracted using the traditional xanthogenate technique (Tillett and Neilan, 2000). For the partial amplification of the 16S rRNA gene, a forward primer (359F, 5'-GGG GAA TYT TCC GCA ATG GG-3') and a reverse primer (781R, 5'-GAC TAC TGG GGT ATC TAA TCC CAT T-3') were used (Nübel et al., 1997). The 16S rRNA amplification was performed using 25µl aliquots containing 30–50µg DNA template, 200µM dinucleotide triphosphates, 0.4µM of forward and reverse primers, 1 U/µl Taq Polymerase and 1.5µM MgCl2, (BioRad, DNA Engine, Peltier Thermal Cycler). The reaction mixture was incubated in a Thermal cycler for DNA amplification (Singh P, et al., 2015). The amplified products were sequenced by Sanger’s method and the obtained sequence was compared with the NCBI database using BLAST tool. MEGA 11 software was used for making maximum likelihood tree for phylogenetic analysis (Guindon and Gascuel, 2003).

### Experimental setup for study

The culture of an early exponential growth phase was harvested throughout centrifugation at 3158 g for 10 min, and the obtained cell pellets were used to make an artificial mat on Petri dishes (90 mm in diameter). We employed two practical dehydration methods for dehydration treatment: the cyanobacterial mat was exposed to laminar airflow for air drying (AD) in the first, and the cyanobacterial mat was placed in a desiccation chamber using calcium chloride fused for desiccation drying (DD) in the second. At various dehydration treatments, including 0, 6, 12, 24, 48, and 96 hours, an *N. calcicola* BOT1 mat was harvested from each dehydrated plate and used for physicochemical analysis. Each of the experiments was carried out in three biological replicates.

### Relative water content

Relative water content was calculated using the following equation:


(% of water) = (FW − DW)/FW 100%

Here, FW denotes the fresh weight of cyanobacteria samples and DW denotes the dry weight determined after chemical drying and air drying in the laminar flow.

### Evaluation of chlorophyll fluorescence (ChlF)

Through the study of ChlF parameters using the pulse-amplitude modulation (PAM-2500, Walz, Germany), the impact of dehydration stress on photosystem II (PSII) and the redox potential of the photosynthetic electron transport chain was evaluated *in-vivo* (Ogawa et al., 2017). As a non-destructive indicator of photosynthetic activity, ChlF was used, and a variety of photosynthetic metrics were used to gauge the health of the cells under dehydration. The data collecting programme PamWin-3 was used to record fluorescence levels of dehydrated cyanobacterial mats. The PamWin-3 software calculates the minimum fluorescence intensity of dark adopted mats (Fo), maximum fluorescence intensity of dark adopted mats (Fm), maximum electron transport rate (ETRmax), maximum photosynthetic quantum yield of the PSII (Fv/Fm), effective photochemical quantum yield [Y(II)], quantum yield of non-regulated energy dissipation [Y(NO)], quantum yield of regulated energy dissipation [Y(NPQ)], and non-photochemical fluorescence quenching (NPQ). To prevent any energy-dependent quenching, dehydrated cyanobacterial mats were left in the dark for 30 min before observations and actinic light (AC) intensity progressively from 3 to 1469 µmol photons m^-2^s^-1^ in order to analyze quantum yield measurements (Bag et al., 2020).

### Visualization of (exopolysaccharides) EPS

For accurate imaging, Alcian blue dye was used to stain the acidic mucopolysaccharides released by cyanobacterial species. A solution of 3% (w/w) acetic acid and 0.33% (w/w) alcian blue (Chroma-Gesellschaft, Kongen, Germany) was used to stain cyanobacterial mats (Tamaru et al., 2005). The stained mats were washed with DDW to remove excess dye, then it was observed under a light microscope.

### Biochemical composition analysis in dehydrated mats

#### Quantification of photosynthetic pigment

For the estimation of lipid-soluble pigments like chlorophyll-*a* (Chl-*a*), carotenoids, and scytonemin, 20 mg of cyanobacterial mats was subjected to 80% acetone, employing the method of Tandeau de Marsac and Houmard (1988) with slight modifications. The mixture was kept at 4°C overnight for the complete extraction of the pigment in the acetone. Furthermore, the amount of chlorophyll and other compounds was determined by measuring the optical density (OD) at different wave lengths, such as 665 nm for Chl-*a*, 461 nm for carotenoids, and 384 nm for scytonemin, and the absorbance at 750 nm was subtracted to account for light scattering (Hirschberg and Chamovitz, 1994). The remaining cell pellets were mixed with DDW and used for the extraction of water-soluble phycobiliproteins using the freeze-thaw method (Bennett and Bogorad, 1973). The supernatant was collected by centrifugation, and the OD of different phycobiliproteins was determined spectrophotometrically at 562, 615, and 652 for phycocyanin (PC), phycoerythrin (PE), and allophycocyanin (APC), respectively. All the pigments were evaluated using the following the standard formulas:


$$\text{Chl}-a\left(µ\text{g}/\text{mg}\right)\;=\;\left({\text{A}}_{665}\text{nm}-{\text{A}}_{750}\text{nm}\right)\;\times 13.9$$

$$\text{Carotenoids }\left(µ\text{g}/\text{mg}\right)\;=\;\left[\left({\text{A}}_{461}\text{nm}-{\text{A}}_{750}\text{nm}\right)\;–\;0.046\times \;\left({\text{A}}_{665}\text{nm}-{\text{A}}_{750}\text{nm}\right)\right]\;\times \;4$$

$$\text{Scytonemin }\left(µ\text{g}/\text{mg}\right)\;=\;\left(1.04\;{\text{A}}_{384}\text{nm }-\;0.79\;{\text{A}}_{663}\text{nm }-\;0.27\;{\text{A}}_{490}\text{nm}\right)\;\times \text{ V}$$

$$\text{PE }\left(µ\text{g}/\text{mg}\right)\;=\;\left\{{\text{A}}_{562}-\;\left(2.41-\text{ PC}\right)\;-\;\left(0.849\;-\text{ APC}\right)\right\}/9.62$$

$$\text{PC }\left(µ\text{g}/\text{mg}\right)\;=\;\left({\text{A}}_{615}-\;0.474\;\times \;{\text{A}}_{652}\right)/5.34$$

$$\text{APC }\left(µ\text{g}/\text{mg}\right)\;=\;\left({\text{A}}_{652}-\;0.208\;\times \;{\text{A}}_{615}\right)/5.09$$

#### Quantification of protein, carbohydrate, and lipid

The total protein content of the dehydrated cyanobacterial mats was quantified using the traditional colorimetric method followed by the standard protocol of Lowry et al. (1951). In brief, 10 mg cyanobacterial mats were first treated with 1 ml of reagent-A (0.1 N NaOH) and kept in a water bath for 30 min. The mixture was then agitated with 2 ml of reagent-B (2M Na_2_CO_3_ and 0.5 M CuSO_4_.5H_2_O in 1M sodium potassium tartrate) for 30 min at room temperature. Then, a further 0.5 ml of 1N folin-ciocalteu reagent (FCR) was mixed and stored for 20 min at room temperature. The development of a blue color confirmed the presence of protein, and their concentration was measured by taking the optical density at 650 nm. During protein estimation, bovine serum albumin (BSA) was used to prepare the standard curve.

Total carbohydrate was quantified using the anthrone method (Loewus, 1952). In brief, 10 mg cyanobacterial mats of each treatment were agitated with 1 ml of 1N NaOH and stored for 25 min in the boiling water bath. The mixture was crushed using a mortar and pestle to obtain a crude homogenate, which was centrifuged at 3158 g for 10 min. Further, 100 µL of each supernatant was used to evaluate the total amount of carbohydrate. The anthrone reagent was prepared in 200 mL of chilled 95% H_2_SO_4_ by dissolving 400 mg anthrone. Then, 1 mL of the sample (100 µL cyanobacterial supernatant + 900 µL DDW) was mixed with 4 mL of freshly prepared anthrone reagent and incubated at room temperature for 15 min. The reaction mixture was placed in the preheated water bath for 15 min, and instant ice cooling was conducted for 5 min to arrest the reaction. The absorbance of each sample was measured at 625 nm (Dubois et al., 1956). A standard curve of glucose was prepared and used for the quantification of the carbohydrate content in dehydrated mats.

The total lipid content was extracted from 50 mg of dried cyanobacterial mat using a slightly modified version of Bligh and Dyer’s protocol (Bligh and Dyer, 1959). The water, methanol, and chloroform mixture were used in a 2:1:2 ratio for the extraction of total lipids from dehydrated samples. Cyanobacterial mats were mixed with the above-mentioned solution and vortexed for 10 min. The mixture was sonicated for 5 min with 30 seconds on and 30 seconds off pulse, at a frequency of 20 kHz. The whole slurry was centrifuged at 12633 g for 15 min, and the lower organic phase was transferred into a previously weighed tube and evaporated at 40°C using the SpeedVac vacuum concentrator. The total lipid content in the mats was calculated using the following equation:


% Lipid content = lipid weight (mg)×100/ dried mat weight (mg)

#### Quantification of osmoprotectant

Trehalose content in dehydrated mats was measured according to the protocol of Lillie and Pringle (1980). In brief, 20 mg of dried mats was mixed with 1 ml of 0.5 M trichloroacetic acid and stored for 1 h at room temperature. Then, the mixture was crushed and centrifuged at 12633 g for 15 min. The subsequent procedure was similar to carbohydrate quantification. The turbidity of the supernatant was recorded at 625 nm, and its amount was expressed as μ mole/mg DW using the trehalose calibration curve.

Proline contents were measured following the approach of Bates et al. (1973). With the aid of a mortar and pestle and 3% (w/v) sulfosalicylic acid, 20 mg of dried mats was homogenized and left at room temperature for 24 h. The homogenate was centrifuged for 20 min at 12633 g and the collected supernatant was then treated with acetic acid and ninhydrin. The process was stopped by submerging the tubes in freezing water after the mixture had been boiling for two hours.

Toluene was used for the final extraction of proline (µ mole/mg DW) and also as a reference; an equal amount of toluene was taken, agitated with cooled solution, and kept for layer separation. Lower pink toluene solution was collected in a separate test tube, and the OD was measured at 520 nm.

#### Quantification of stress biomarkers

MDA was quantified using 2-Thiobarbituric acid (TBA) and indicated as equivalent to lipid peroxidation (Heath and Packer, 1968). In a 5% trichloroacetic acid (TCA) solution, cyanobacterial mats were homogenized using a mortar and pestle. Centrifuging homogenate mixes took place for 10 min at 12633 g. After being heated to boiling temperature for 20 min, 1 ml of the supernatant was combined with 1 ml of 0.65 M TBA (i.e., TBA produced in a 20 M TCA solution). The mixture was centrifuged at 12633 g for 10 min after cooling in ice-cold water. At 450, 532, and 600 nm, measurements of the supernatant’s absorbance were made. The following equation (Chokshi et al., 2015) was used to calculate the MDA concentration:


$$\text{MDA }\left(µ\text{ mol}/\text{mg DW}\right)\;=\;\left[6.45\;\times \;\left({\text{A}}_{532}\text{nm }–\;{\text{A}}_{600}\text{nm}\right)\right]\;–\;\left[0.56\;\times \;{\text{A}}_{450}\text{nm}\right]/\text{dry weight }\left(\text{mg}\right)$$

Cyanobacterial mats were crushed in a 0.1 M TCA solution to determine the amount of H_2_O_2_. Then, 0.5 ml of supernatant was added to the reaction mixture, which contained 0.5 ml of 0.1 M phosphate buffer saline (pH 7.0) and 1 ml of 1 M, KI solution. Further absorbance was taken at 390 nm and the amount of H_2_O_2_ was shown as µ mol/mg DW (Velikova et al., 2000).

#### Quantification of non-enzymatic antioxidants

To evaluate the total flavonoid (TFC) and phenolic content (TPC), the samples were crushed in the 90% acetone solution. The TFC of dehydrated mats was measured using aluminum chloride (AlCl_3_) (Ordonez et al., 2006), and 1 ml of the extract was further mixed with 1 ml of 2 M AlCl_3_; the solution was gently agitated and stored for 2 hours at room temperature. The colour of the mixture was changed and then the OD of the samples were measured at 420 nm. TFC was expressed as µg quercetin (QE)/mg DW.

The TPC of dehydrated mats was measured colorimetrically using the FCR following the standard protocol of Singleton et al. (1999). A total of 0.5 ml supernatant, 1 ml of 2 M Na_2_CO_3,_ and 0.5 ml of 1N FCR were mixed and maintained 5 ml final volume using DDW; the mixture was then heated until blue color developed. The mixtures were allowed to cool at room temperature before measuring absorbance at 760 nm. TPC was expressed in µg gallic acid equivalent (GAE)/mg DW.

### Vital staining of cells with Fluorescein Diacetate (FDA) and Propidium Iodide (PI)

For the determination of the effect of dehydration treatment on the viability of cells, we used the fluorescence microscopy technique with vital staining dyes, i.e., FDA and PI. FDA is a non-fluorescent, uncharged, lipid-soluble dye that is hydrolyzed to fluorescein by non-specific intracellular esterases after uptake and stains live cells (Tamaru et al., 2005).

Since dead and damaged cells lack an unbroken cell membrane, PI, a red fluorescent cell viability dye, can only enter damaged or dead cells (Deligeorgiev et al., 2009). Once inside, it intercalates between the two to bind to DNA. The FDA stock solution was made by combining 3 mg of FDA with 1 ml of cold acetone and storing it at -20°C, while the PI stock solution was made by combining 1 mg of PI with 1 ml of 8X phosphate-buffered saline (PBS) and storing it at 4°C. Using an FDA excitation filter at 480 nm and a 585 nm emission filter at the green channel, and a PI excitation filter at 493 nm and a 636 nm emission filter at the red channel, it was possible to see the live-dead cells (Tamaru et al., 2005).

### Extraction of metabolites

Metabolites were extracted from control and 96 h DD mats using HPLC-grade methanol (Thanh Doan et al., 2000). Methanol-filled flasks with mats were chilled overnight at 4°C then further centrifuged at 10,000 g for 25 minutes, and supernatants were collected separately. Then, the supernatants were dried through evaporation and redissolved in methanol of HRMS grade.

UHPLC-HRMS analysis of extracts was carried out using an Orbitrap Eclipse Tribrid Mass Spectrometer USA, from the Central Discovery Centre (CDC), Banaras Hindu University BHU, Varanasi, Uttar Pradesh, India. UHPLC was used to separate small molecules chromatographically (COMPOUND DISCOVERER 3.3.2.31). Here, a three-solvent system was used as the mobile phase: Solvent A was water with 0.1% formic acid; Solvent B was 100% acetonitrile with 0.1% formic acid; and Solvent C was 100% methanol with 0.1% formic acid. The metabolites were separated using a GOLD C18 selectivity HPLC column (inner diameter 2.1 mm, length 100 mm and particle size 1.9 µm). The injection volume was 5 µl, the run time was 30 min., and the flow rate was 0.3 ml/min. The column outlet was connected to a mass spectrometer *via* H-ESI (electrospray ionization). Both negative and positive modes of H-ESI were used to ionize the compounds that were channelized using Orbitrap. To identify the likely compounds present in the extract, the MS spectra for the analyzed samples were compared to those from the Predicted Compositions, mzCloud Search, ChemSpider Search, and MassList Search databases.

### Statistical analysis

All the data are presented as the mean of three replicates, and to assess the significant difference, data were statistically analyzed using a one-way analysis of variance (ANOVA) with a significance level of p<0.05 (SPSS 16.3 statistics version, Chicago, IL, USA).

## Results and discussions

### Isolation, identification, and growth behavior of cyanobacterial isolate

**Figure 1:**
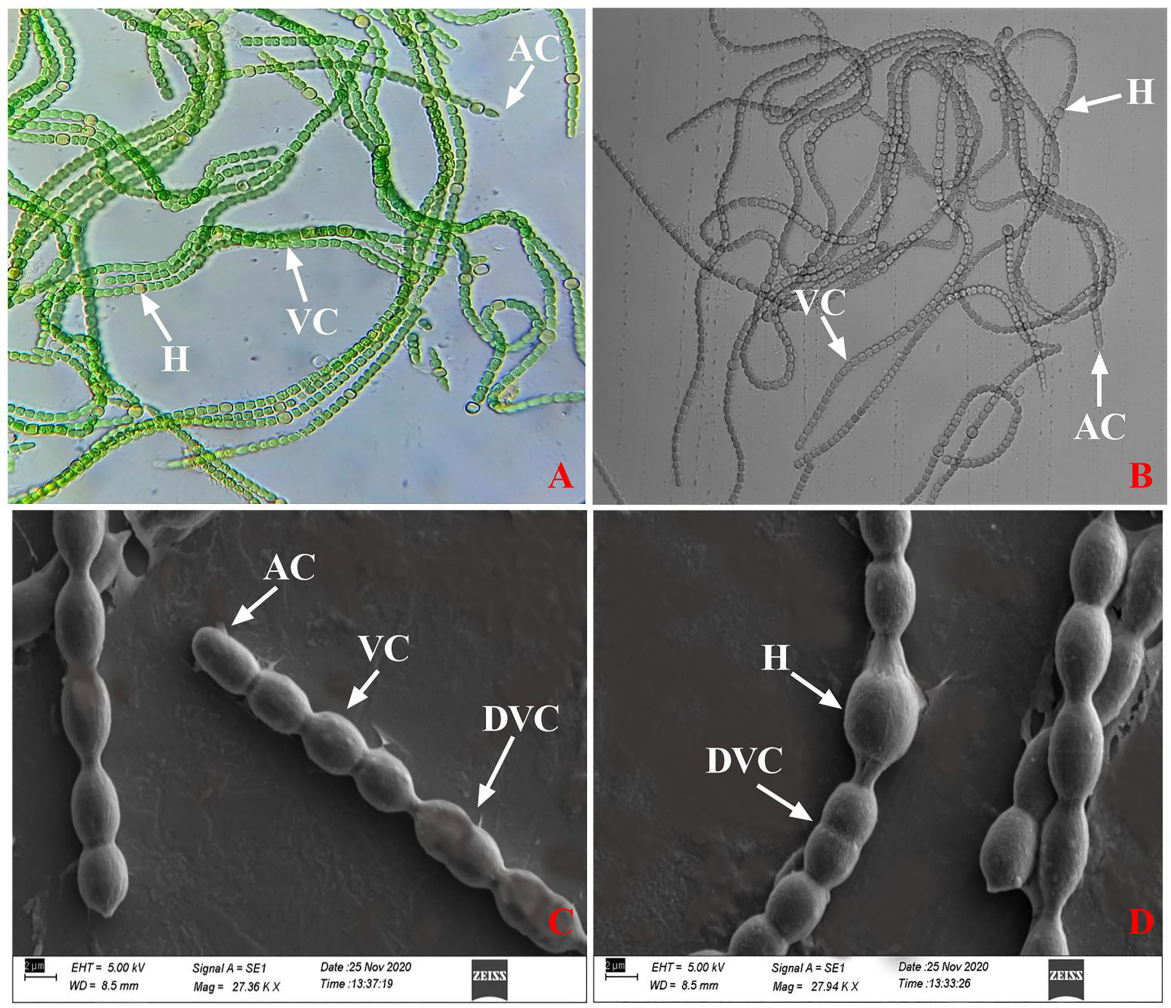
**(A, B)** Light and brightfield microscopic image and **(C, D)** SEM image of *N. calcicola* BOT1. Were, AC: apical cell, H: heterocyst, VC: vegetative cell, DVC: dividing vegetative cell.

To confirm the identity of cyanobacterial isolates, light and brightfield microscopy (**Figures 1A, B**) was used first, followed by morphological and molecular characterization to further confirm the identity of the isolates.

The presence of a filamentous body with moniliform vegetative cells interrupted with pale yellow intercalary heterocysts and a dome-shaped terminal cell indicated that isolated cyanobacteria are a *Nostoc* species (Desikachary, 1959; Celis-Pla et al., 2021; Usmonkulova et al., 2022). Furthermore, SEM was used to confirm the morphology of isolated cyanobacteria, and all of the present micrographs showed similarities in shape, size, and surface morphology with the previously reported *Nostoc* (Komárek, 2013; Tiwari et al., 2019) (**Figures 1C, D**).

**Figure 2:**
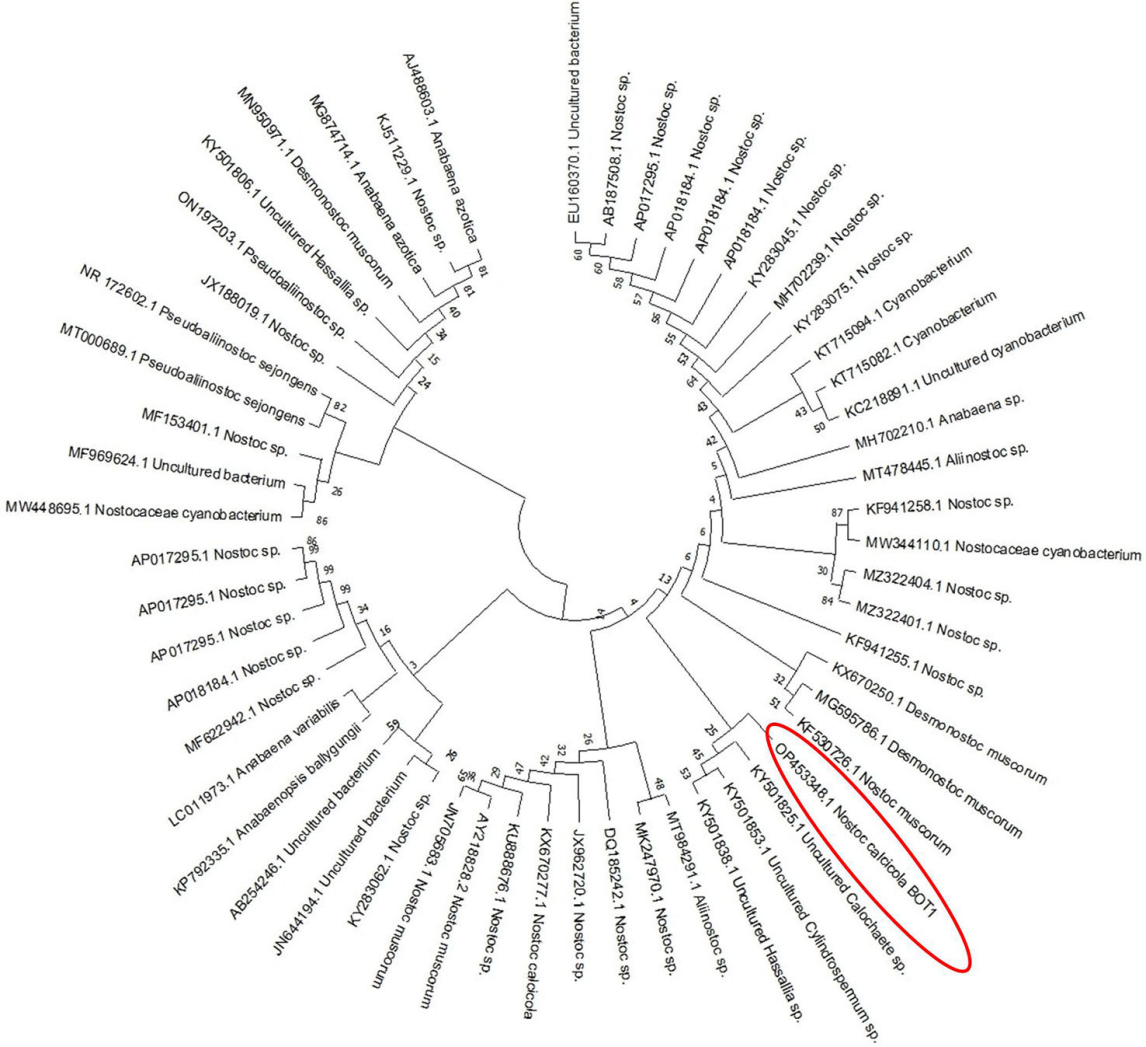
Phylogenetic tree of *N. calcicola* BOT1, based on 16S rRNA gene sequence.

The molecular identification of the cyanobacterial strain was found using 16S rRNA gene sequencing. The maximum likelihood phylogenetic analyses (**Figure 2**) of 16S rRNA showed a similarity of 99% with the closest species, *Nostoc calcicola*. Furthermore, the gene sequence was deposited in the NCBI gene bank database with Gene-Bank Accession No. OP453348. However, the best growing circumstances were discovered to be at 14:10 h light-dark, with a photoperiod of 55 µmole m^-2^s^-1^ light intensity and a temperature of 25°C.

### Effect of dehydration on morphological changes and EPS production

**Figure 3:**
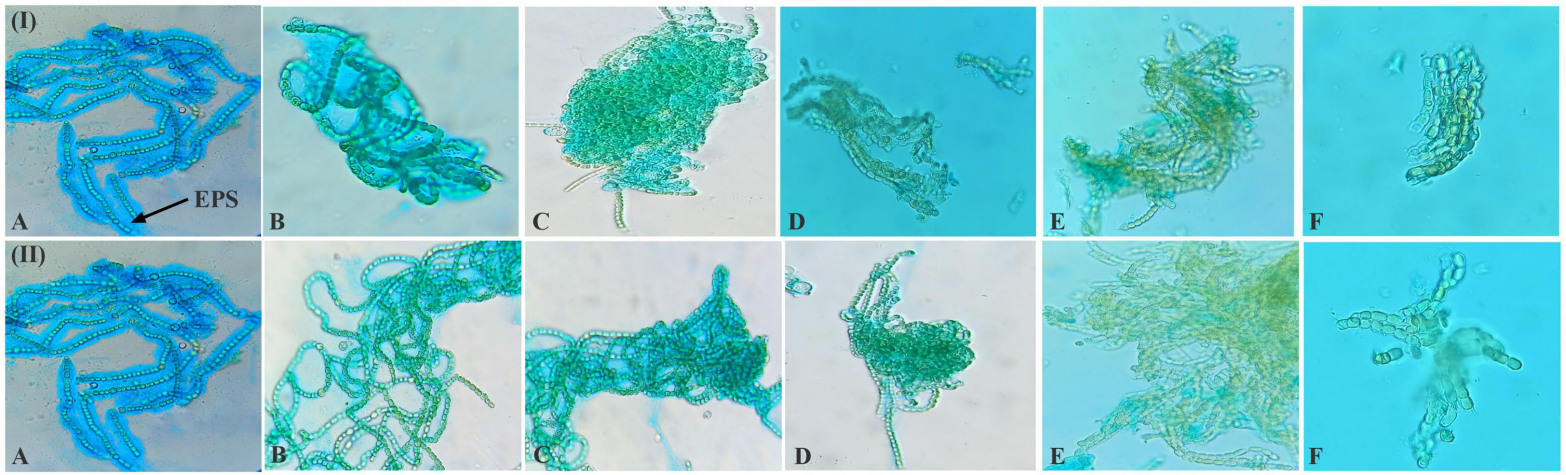
Light microscopic photographs (at 20X magnification) of *N. calcicola* BOT1 at different durations of dehydration treatment. Mucopolypeptide-binding with alcian blue (non-fluorescence dye) was used to stain cyanobacterial mats. (I) air-dried mats and (II) desiccator-dried mats in which, **(A)** indicates control, and **(B–F)** denote dehydration treatments for 6, 12, 24, 48, and 96 hours, respectively.

The EPS of cyanobacteria is unique in its constituents, i.e., its protein, nucleic acid, and lipid content (Costa et al., 2018). EPS has the ability to retain water, thus protecting the photosynthetic apparatus by slowing the rate of desiccation (Raanan et al., 2016; Benard et al., 2019). Since EPS is cohesive, it can shield soil from water and wind erosion, preserve the fertility of soil, and increase water retention (Faist et al., 2017). In the study, dehydration causes morphological changes and affects EPS secretion around the filaments in *N. calcicola* BOT1 during dehydration (**Figure 3**). The released EPS was stained using alcian blue dye, and it reached its maximum in the control and in 6 h AD and DD dehydrated mats. This experiment was done initially with cyanobacterial filaments, which were not wet beforehand. All microphotographs were taken at the same magnification. With an increase in dehydration duration, there was a rapid decrease in EPS production.

The filament length and width of cells and EPS secretion varied with the change in the duration of dehydration in both AD and DD mats (**Figure 3**). This result demonstrated that *N. calcicola* BOT1 filaments became shorter and more compact under dehydration conditions in both AD and DD mats and also formed a compact mat by aggregating their filaments. A similar observation was also reported by Feng et al. (2012) in *Nostoc flagelliforme*. This capability of *N. calcicola* BOT1 makes it suitable for biocrust formation.

### Effect of dehydration on photosynthetic efficiency

In semi-arid and dry environments, where cyanobacteria are frequently subjected to periods of desiccation, water availability is a significant factor impacting cyanobacterial growth (Chaves et al., 2003). Numerous metabolic functions, including photosynthesis, are adversely impacted by desiccation stress. For instance, a lack of water harms the fundamental structure, which prevents the uptake of carbon and harms the photosynthetic machinery of photosynthetic organisms (Golldack et al., 2011).

The highest quantum yield of PSII is indicated by Fv/Fm (Kitajima and Butler, 1975). However, with the RWC dropping from 60 to 80% as a result of dehydration, Fv/Fm decreased quickly. Cyanobacteria were classified as sensitive, semi-tolerant, and desiccation-tolerant by Raanan et al. (2016) based on measurements of oxygen evolution rate and Fv/Fm in response to light stress. Due to the existence of EPS, which serves as an exterior barrier against desiccation in *Nostoc* sp., they believed that *Nostoc* sp. came under the desiccation-tolerant group (Shirkey et al., 2000).

**Figure 4:**
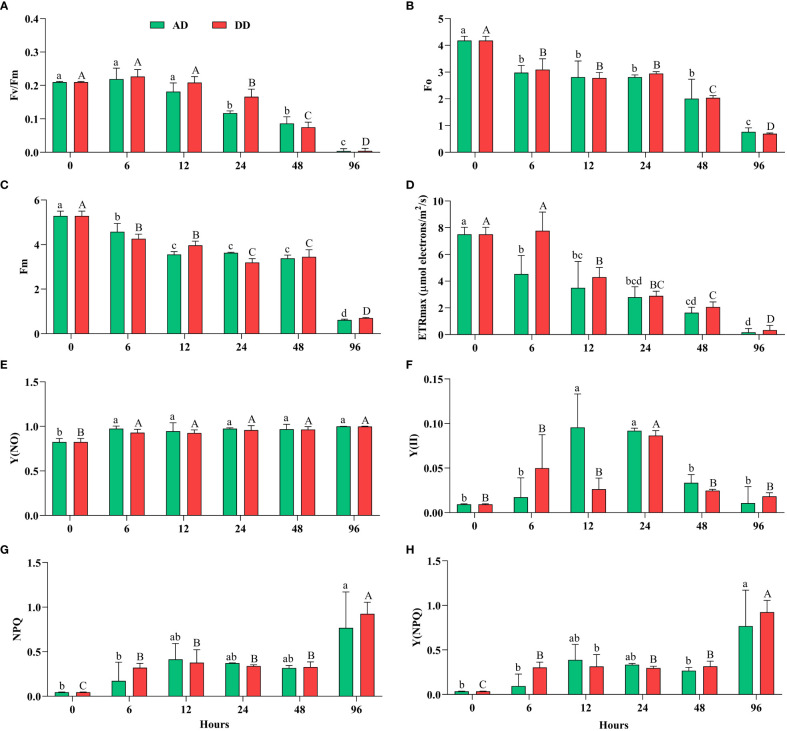
Effect of different durations of dehydration on the photosynthetic efficiency of *N. calcicola* BOT1. Different superscript alphabet letters on the values indicated a significant difference (p<0.05) between the mats of different durations of dehydration treatments. **(A)**. (Fv/Fm) is the maximum photosynthetic quantum yield of PS II, **(B)**. (Fo) is the minimum fluorescence, **(C)**. (Fm) maximum fluorescence, **(D)**. (ETRmax) maximum electron transfer rate, **(E)**. [Y(NO)] non-regulated energy dissipation quantum yield of PS II, **(F)**. (YII) amount of energy used in photochemistry by PS II, **(G)**. (NPQ) non-photochemical fluorescence quenching, and **(H)**. Y(NPQ) regulated energy dissipation quantum yield of PS II. The error bars on the histograms indicate the standard error of the mean values of three biological replicates.

All oxygenic photosynthetic organisms have been extensively studied using ChlF, a delicate mirror of photosynthesis (Housman et al., 2006; Baker and Oxborough, 2004; Baker, 2008). Fv/Fm can offer a quick and easy technique to determine whether or not cyanobacterial colonies were subjected to stressful conditions (Henriques, 2009). When *N. calcicola* BOT1 cells were dehydrated for various amounts of time (6-96 hours) Fv/Fm, Fo, Fm, YII, and ETRmax significantly (p<0.05) decreased, while NPQ, Y(NO), and Y(NPQ) increased, in comparison to the control (**Figure 4**).

This implies a significant impact of dehydration on the photosynthetic activity of the cyanobacterial isolate *N. calcicola* BOT1. The minimum Fv/Fm was found in 96 h AD and DD mats, which was (0.0040 ± 0.004) and (0.0043 ± 0.004), respectively. During Fv/Fm evaluation, cyanobacteria cells were subjected to subsequent dehydration for 6 to 96 h; there was a significant maximum drop in 96 h compared to the control, 52.5 fold in AD and 48.8 fold in DD mats (**Figure 4A**). In this study, Fv/Fm significantly decreased in both AD and DD after 12 h of dehydration treatment (**Figure 4A**).

Our results indicate that Fv/Fm was maintained during 0-12 h of dehydration treatment under both AD and DD conditions; however, this reduced when the RWC decreased below 60%. Data for *N. calcicola* BOT1 (**Figure 4**), however, indicate a fast decline of Fv/Fm values at the RWCs below 60%, suggesting a sensitivity of *N. calcicola* BOT1 primary photosynthetic processes to desiccation (Alonso, 2018). This behavior simply depended on the degree of dryness and was unaffected by the drying rate. Disregarding the potential for dried, stabilized Chl-*a* to absorb red light during the PAM test, the observed decrease in Fv/Fm, which did not approach zero even after 96 hours of dehydration, may conceivably reflect a very minimal but continuing function of the photosynthetic apparatus.

In 96 h dehydrated mats, Fo and Fm were significantly (p<0.05) lower than the controls; they were (0.766 ± 0.086) and (0.616 ± 0.019) in AD, and (0.693 ± 0.018) and (0.700 ± 0.019) in DD mats, respectively (**Figures 4B, C**). The decreased Fo (5.45 and 6.03 fold) and Fm (8.58 and 7.55 fold) in AD and DD mats indicated that both PSI and PSII light-harvesting complexes and the reaction center were inactive. These ratios, according to Heber et al. (2007), are an indication of variations in the degree of photoprotection, which ought to be greater in severely dehydrated cyanobacterial mats than in mildly dehydrated ones. Only very modest residual charge separation levels were seen in AD and DD mats at the end of the dehydration under the circumstances of our experiment. In desiccated mats, the Fo values were considerably low (P<0.05), indicating higher levels of light energy dissipation in cyanobacterial mats that had completely dried out.

ETRmax followed the same pattern as Fv/Fm and decreased gradually up to 46.8 fold in AD and 22.7 fold in DD mats after 96 h of dehydration **Figure 4D**). It is believed that this stoppage of electron transport is an acclimatory reaction to desiccation (Fukuda et al., 2008). Dehydration significantly decreased the ETRmax, which suggests a decrease in the PSII reaction center, as seen by the light curve. The change from cyclic to sparse linear electron flow is what causes the reduced ETRmax. This conversion of electron flux protects the PSII from excessive activation energy of electrons (Fukuda et al., 2008).

Furthermore, NPQ and Y(NPQ) (**Figures 4G, H**) increased more than the control at 96 h in both AD and DD mats, (0.767 ± 0.232), (0.768 ± 0.232), and (0.925 ± 0.074), (0.924 ± 0.075), i.e., 17.0, 21.3, 20.5, and 25.6 fold, respectively. The rise in the nonphotochemical quenching (NPQ) value provided additional proof of physiological stress (Deng et al., 2014). By dispersing the extra energy as heat, NPQ prevents damage to the photosynthetic reaction center (White et al., 2011). Carotenoid conversion into photo-protective pigments under stress is correlated with a high NPQ value (Boussiba, 2000).

The decrease in Fo and Fm and the increase in NPQ and Y(NPQ) after 96 hours of dehydration suggest an enhancement in energy *via* the xanthophyll cycle (García-Plazaola et al., 2007; Jahns and Holzwarth, 2012). Y(NO) increased significantly in all the treatments, but there was an insignificant difference found in AD and DD mats (**Figure 4E**), and it was (0.999 ± 0.000) and (0.997 ± 0.002), i.e., 1.21 and 1.20 fold, respectively. As previously mentioned, an increase in Y(NPQ) denotes an effort to release excess energy, whereas an increase in Y(NO) denotes excess energy fluxes that are out of control and may result in photodamage to *N. calcicola* BOT1 (Kramer et al., 2004). Severe water stress caused a decrease in Y(II) and an increase in Y(NO), which indicates that dehydration stress increased the fraction of non-reducing and “closed” PS II reaction centers. However, when the internal water content reached 60-40%, Y(II) increased, but increasing the duration of dehydration reduced the Y(II) value (Deng et al., 2014). The lower Y(II) values from the 48-96 h dehydration treatments suggest that water content below a certain point has a greater impact on PS II activity. The quenching parameter Y(NO) increased as dehydration treatment increased. According to Pfündel et al. (2008), a rise in Y (NO) shows an enhancement in the proportion of “closed” PSII centers and PSII’s inability to defend itself from photodamage. In this experiment, Y (NPQ) was significantly affected by the dehydration treatment. NPQ, which depends on the presence of carotenoids, protects PS II against stress-related damage. Dehydration treatment reduced carotenoids content after 48 hours in both AD and DD mats (**Table 1**), indicating that NPQ was not protecting PS II from dehydration-induced photoinhibition by regulating energy dissipation Y(NPQ) (Deng et al., 2014).

**Table 1:** Effects of different durations of dehydration treatment on photosynthetic pigments of *N. calcicola* BOT1.

Duration of treatment	Chlorophyll-*a* (µg/mg DW)		Carotenoids (µg/mg DW)		Scytonemin (µg/mg DW)		Chl-*a*/Caro ratio (µg/mg DW)		Chl-*a*/Scyt ratio (µg/mg DW)	
	AD	DD	AD	DD	AD	DD	AD	DD	AD	DD
0	2.81 ± 0.00^a^	2.81 ± 0.00^A^	2.85 ± 0.00^d^	2.85 ± 0.00^E^	0.10 ± 0.00^e^	0.10 ± 0.00^D^	0.98 ± 0.00^a^	0.98 ± 0.00^A^	28.18 ± 0.67^a^	28.18 ± 0.67^A^
6	2.47 ± 0.23^ab^	2.75 ± 0.08^A^	2.71 ± 0.00^e^	2.26 ± 0.00^F^	0.25 ± 0.01^d^	0.30 ± 0.03^C^	0.76 ± 0.00^c^	0.83 ± 0.00^C^	3.07 ± 0.00^d^	3.30 ± 0.00^C^
12	2.46 ± 0.05^ab^	2.53 ± 0.16^AB^	2.05 ± 0.00^f^	3.34 ± 0.00^B^	0.43 ± 0.00^b^	0.49 ± 0.01^B^	0.90 ± 0.00^b^	0.80 ± 0.00^D^	4.25 ± 0.00^d^	4.58 ± 0.02^BC^
24	2.20 ± 0.05^b^	2.23 ± 0.03^B^	3.11 ± 0.00^b^	3.00 ± 0.00^D^	0.39 ± 0.01^c^	0.46 ± 0.00^B^	0.73 ± 0.00^d^	0.86 ± 0.00^B^	8.72 ± 0.02^b^	5.64 ± 0.00^B^
48	2.15 ± 0.03^b^	2.27 ± 0.05^B^	3.36 ± 0.00^a^	3.41 ± 0.00^A^	0.43 ± 0.00^b^	0.51 ± 0.00^B^	0.70 ± 0.00^e^	0.78 ± 0.00^E^	5.49 ± 0.00^c^	5.19 ± 0.01^B^
96	2.04 ± 0.00^b^	2.20 ± 0.00^B^	2.93 ± 0.00^c^	3.28 ± 0.00^C^	0.51 ± 0.00^a^	0.61 ± 0.00^A^	0.69 ± 0.00^f^	0.67 ± 0.00^F^	3.98 ± 0.00^d^	3.57 ± 0.00^CD^
Phycobiliproteins										
	Phycocyanin (µg/mg DW)		Phycoerythrin (µg/mg DW)		Allophycocyanin (µg/mg DW)		Total phycobiliproteins (TPBPs) (µg/mg DW)		TPBP/Chl-*a* (µg/mg DW)	
	AD	DD	AD	DD	AD	DD	AD	DD	AD	DD
0	7.02 ± 0.01^a^	7.02 ± 0.01^A^	1.59 ± 0.01^a^	1.59 ± 0.01^A^	3.96 ± 0.03^a^	3.96 ± 0.03^A^	12.58 ± 0.08^a^	12.58 ± 0.08^A^	4.46 ± 0.01^a^	4.46 ± 0.01^A^
6	6.73 ± 0.16^b^	7.01 ± 0.58^A^	1.11 ± 0.01^b^	1.17 ± 0.09^B^	2.86 ± 0.01^b^	3.97 ± 0.00^A^	10.71 ± 0.17^b^	12.16 ± 0.52^A^	4.39 ± 0.38^a^	4.43 ± 0.34^A^
12	5.18 ± 0.01^c^	5.73 ± 0.11^B^	0.83 ± 0.01^c^	0.85 ± 0.02^C^	2.87 ± 0.01^b^	2.40 ± 0.00^B^	8.90 ± 0.01^c^	8.99 ± 0.11^B^	3.61 ± 0.08^b^	3.57 ± 0.20^B^
24	5.04 ± 0.02^c^	4.16 ± 0.13^C^	0.71 ± 0.03^d^	0.82 ± 0.05^C^	2.23 ± 0.05^d^	2.15 ± 0.02^C^	7.99 ± 0.05^d^	7.14 ± 0.10^C^	3.63 ± 0.07^b^	3.20 ± 0.07^BC^
48	3.44 ± 0.02^e^	2.92 ± 0.09^D^	0.43 ± 0.05^e^	0.77 ± 0.01^C^	2.42 ± 0.05^c^	2.09 ± 0.03^C^	6.29 ± 0.04^e^	5.79 ± 0.09^D^	2.92 ± 0.06^bc^	2.54 ± 0.04^CD^
96	3.94 ± 0.05^d^	2.83 ± 0.02^D^	0.40 ± 0.04^e^	0.40 ± 0.02^D^	1.18 ± 0.04^e^	1.27 ± 0.08^D^	5.53 ± 0.10^f^	4.51 ± 0.08^E^	2.70 ± 0.05^c^	2.04 ± 0.03^D^

Chl-a: Chlorophyll-a, Caro: Carotenoids, Scyt: Scytonemin.

Different letters indicate a significant difference (p<0.05) between the mats of different durations of dehydration treatments. The data given here is the mean of three biological replicates with standard error.

In our investigation, it was shown through the rise in NPQ, Y(NO), and Y(NPQ) (**Figures 4E–H**) in AD and DD mats at 96 h of dehydration compared to the control that the extra energy surpassed the cyanobacteria’s capacity for regulation and could not be properly dissipated, particularly under extreme stress. It might indicate irreversible cell dehydration and impaired metabolism (Kramer et al., 2004; Radermacher et al., 2019).

### Effect of dehydration on pigment

A crucial marker of the oxygenic photoautotrophic nature of cyanobacteria is the presence of photosynthetic pigments, Chl-*a*, and phycobiliproteins. The effect of dehydration stress on the different pigment concentration monitored in *N. calcicola* BOT1 which is represented in **Table 1**. Osmotic stress caused by decreasing water content reduced pigment concentrations. Reduced photosynthetic pigment content could be a sign of oxidative stress caused by decreasing water content and reduced RuBisCo activity because of poor water content that encourages pigment deterioration (Hounslow et al., 2021). Chl-*a* content decreased with increasing dehydration treatment duration. A significant change in Chl-*a* content was observed in 96 h AD (2.042 ± 0.000 µg/mg DW) and DD (2.202 ± 0.000 µg/mg DW) dehydrated mats, which was 1.37 and 1.279 fold less than the control, respectively. Scytonemin content and carotenoid levels both increase concurrently with dehydration treatment duration. Scytonemin concentration was at its maximum in 96 h AD (5.12 ± 0.000 µg/mg DW) and DD (0.615 ± 0.000 µg/mg DW) samples, which was 5.12 and 6.15 fold higher than the control. Carotenoids levels in 48-hour AD and DD samples were (3.366 ± 0.001 µg/mg DW) and (3.414 ± 0.001 µg/mg DW), respectively, which was 1.17 and 1.19 fold higher than the control sample.

Free radicals and singlet oxygen are quenched, protecting cells from reactive oxygen damage under dehydration stress, which is thought to be the cause of the rise in carotenoids (Tamura and Ishikita, 2020). Carotenoids serve as antioxidants in addition to harvesting light in the blue-green spectrum, and under various stress circumstances, their concentration rises along with a corresponding decline in Chl-*a* (Stamatakis et al., 2014; Zakar et al., 2017). Carotenoids regulate how much energy is transferred from the phycobilisome to the PSII reaction center (Kirilovsky, 2007). Zeaxanthin, a type of carotenoid, is crucial for photosynthetic organisms to respond quickly to stress. It has been thoroughly demonstrated that zeaxanthin binding to the PSII antenna system aids in the dissipation of extra chlorophyll-excited states and the scavenging of oxygen radicals (Ballottari et al., 2014; Tian et al., 2017).

The Chl-*a*/carotenoids ratio decreased significantly with increasing dehydration and reached its minimum in 96 h AD and DD mats, which was (0.695 ± 0.000 µg/mg DW) and (0.670 ± 0.000 µg/mg DW), respectively. This result is very similar to Tammam et al. (2011). The Chl-*a*/scytonemin ratio was very similar to the Chl-*a*/carotenoids ratio; both decreased during subsequent dehydration treatment compared to the control.

**Figure 5:**
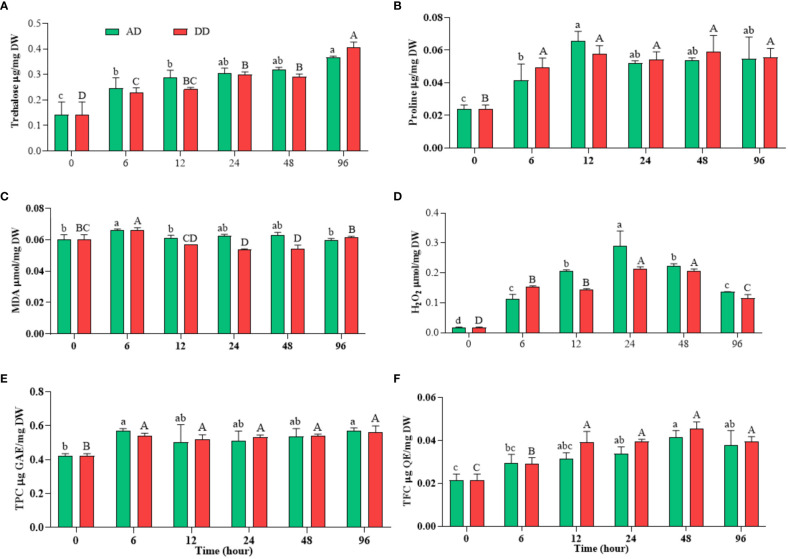
Effects of different durations of dehydration on osmoprotectants (**(A)** trehalose and **(B)** proline), stress biomarkers (**(C)** MDA and **(D)** H_2_O_2_), and non-enzymatic antioxidant (**(E)** TPC and **(F)** TFC) of *N. calcicola* BOT1. Different superscript alphabet letters on the bar indicated a significant difference (p<0.05) between the mats of different durations of dehydration treatments. The error bars on the histograms indicate the standard error of the mean values of three biological replicates.

So, a change in the Chl-/carotenoids ratio serves as a helpful physiological measure that may be utilized to evaluate how the stressor affects ROS levels (Zakar et al., 2017). Low Chl-*a*/carotenoids ratios during dehydration stress (**Table 1**) indicated that dehydrated mats had significant ROS levels, which were validated by MDA and H2O2 measurements (**Figure 5**).

Phycobiliproteins are characteristic-colored pigments found in cyanobacteria and red algae. They act as the light-harvesting complex of PSII during photosynthesis. The PC (7.027 ± 0.015 µg/mg DW) and PE (7.958 ± 0.079 µg/mg DW) content were highest in the control compared to dehydration durations. PC decreased significantly (p<0.05) in 96 h AD and DD mats, which were (3.942 ± 0.054 µg/mg DW) and (2.837 ± 0.024 µg/mg DW), respectively, i.e., 1.78 and 2.47 fold lower than the control (**Table 1**). PE has a similar transcription pattern to PC, which is (2.044 ± 0.126 µg/mg DW) and (2.009 ± 0.081 µg/mg DW), i.e., 3.89 and 3.96 fold lower than the control in 96 h AD and DD mats, respectively. APC was 3.36, 3.11 fold lower in 96 h AD and DD mats than the control. Our results indicate that total phycobiliproteins (TPBP) were at their maximum in the control (18.954 ± 0.111 µg/mg DW), but their content decreased significantly (p<0.05) in AD and DD mats at 96 h of dehydration treatment. The TPBP/Chl-*a* ratio exhibited the same trend as TPBP and decreased significantly in AD and DD mats after 6, 12, 24, 48, and 96 hours of dehydration treatment (**Table 1**). The results indicate that severe dehydration reduces their synthesis and promotes degradation under conditions of water stress.

### Dehydration-dependent accumulation of protein, carbohydrate, and lipid contents

**Figure 6:**
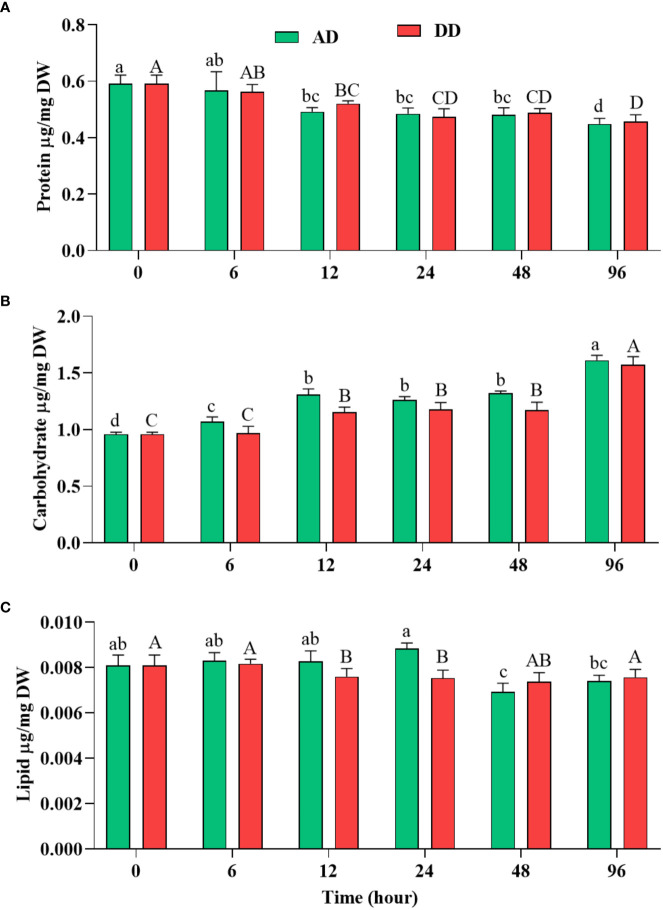
Effect of different duration of dehydration on different biochemicals of *N. calcicola* BOT1. Different superscript alphabet letters on the bar indicate a significant difference (p<0.05) between the mats of different durations of dehydration treatments. **(A)** protein, **(B)** carbohydrate, and **(C)** lipid. The error bars on the histograms indicate the standard error of the mean values of three biological replicates.

Primary metabolites like carbohydrates, proteins, and lipids are extensively synthesized and utilized during the growth phase. Protein content is the marker of growth and its concentration decreased significantly (p<0.05) during dehydration treatment (**Figure 6A**). Protein content was highest in the control, at (0.592 ± 0.017 µg/mg DW), and was at its minimum in 96 h dehydrated mats, at (0.449 ± 0.011 µg/mg DW) in AD mats and (0.457 ± 0.014 µg/mg DW) in DD mats.

The dried mats from AD and DD, which were 1.31 and 1.29 fold less than the control, did not show a significant difference. Notably, high TPBP contents directly correlated with high protein accumulation under control conditions, while TPBP contents accumulated at the lowest levels in 96 h AD and DD mats. Similar results were also obtained in several microalgae under abiotic stress (Fal et al., 2022). Autophagy, degradation, and downregulation of genes may be the primary causes of decreased protein and pigment content in dehydrated cells (Pancha et al., 2015; Chokshi et al., 2017). The reduction in protein content was very similar, with photosynthetic efficiency and different pigment content.

Under dehydration stress, carbohydrate content increased significantly (p<0.05) and its use by cells during prolonged stress conditions was shown (**Figure 6B**). Carbohydrate quantitively increased depending on the stress levels of cells. Dehydration treatment provided the highest level of support for carbohydrate accumulation compared to the control, in N. calcicola BOT1. (**Figure 6B**). It was significantly (p<0.05) increased in 96 h AD and DD mats, at (1.611 ± 0.025 µg/mg DW) and (1.574 ± 0.040 µg/mg DW), i.e., 1.67 and 1.64 fold greater than the control. Under prolonged stress conditions, cyanobacteria utilized stored carbohydrates as osmoprotectants, like sucrose, trehalose, etc., to ensure homeostasis, maintain osmotic status, and promote adaptability to stress conditions (Tietel et al., 2020).

Abiotic stresses induce the accumulation of lipids in cells, which stabilize the plasma membrane during stressful conditions. Several previous reports have also suggested that lipid content increased during stress conditions due to the conversion of carbohydrates to lipids by several metabolic pathways (Hounslow et al., 2021; Jin et al., 2021). Lipid accumulation during dehydration was measured using the gravimetric method (Feng et al., 2013), which showed a similar percentage of lipid content in all the treatments, at (0.0076 ± 0.000 µg/mg DW) and (0.0075 ± 0.000 µg/mg DW), which were decreased by 1.21 and 1.24 fold in 12 and 24 h DD mats, respectively compared to the control (**Figure 6C**). Our findings suggest that there was no role for lipids in the survival of *N. calcicola* BOT1 under dehydration stress.

### Accumulation of osmoprotectants in response to dehydration

Under the conditions of water scarcity, generally, sugars provide stability of proteins, membranes, and whole cells, but in cases of acute water loss, only disaccharides trehalose and sucrose have the ability to provide protection (Potts, 1994). Desiccation appears to be the catalyst for trehalose buildup as the concentration rises rapidly when the water content falls below a certain level (Yoshida and Sakamoto, 2009). The rate of trehalose generation is greater than the rate of hydrolysis, and it has been hypothesized that the trehalase enzyme is crucial in the accumulation of trehalose. This is accomplished through the particular inactivation of trehalase in situations of water stress, which are characterized by elevated concentrations of cellular solutes (Yoshida and Sakamoto, 2009). In untreated *N. calcicola* BOT1, it was (0.142 ± 0.028 μ mole/mg DW), while with the increased duration of dehydration, its concentration increased and vice versa, reaching its maximum in 96 h AD and DD mats, at (0.366 ± 0.003 μ mole/mg DW) and (0.0407 ± 0.011 μ mole/mg DW), respectively (**Figure 5A**). The trehalose concentration of 96 h AD and DD mats was enhanced 2.577 and 2.86 fold compared to untreated mats, which showed a dehydration response.

In line with these findings, natural *N. commune* colonies do not exhibit trehalose buildup in response to desiccation (Sakamoto et al., 2009), indicating that the control of trehalose metabolism may differ in these *Nostoc* species. Trehalose concentration in *N. calcicola* BOT1 was higher in the dehydrated mats compared to the control, but it is still sensitive to desiccation; this study is also comparable to Sakamoto et al. (2009).

Since most stress proteins are water-soluble, they aid in the process of stress tolerance by keeping cellular structures hydrated. Dehydration stress also enhances proline synthesis. Proline is an essential amino acid that performs a number of functions, including preserving the cytosolic pH, acting as a compatible solute, scavenging ROS, and acting as a chaperone molecule to preserve the integrity of protein structure (Fal et al., 2022). Our results show that *N. calcicola* BOT1 had significantly (p<0.05) more proline than the control (**Figure 5B**). Proline accumulation in 12 h AD and DD mats was (0.065 ± 0.003 μ mole/mg DW) and (0.057 ± 0.002 µ mol/mg DW), which was 2.74 and 2.40 fold greater than the control. Sarker et al. (2018) found similar results in *Amaranthus tricolor* under drought stress. The levels of proline rose during drought stress, while soluble protein decreased under stress conditions (Abid et al., 2018; Barnawal et al., 2019). Glycine, betaine, and proline increased the turgor potential within cells, which enable cyanobacteria to adapt against desiccation stress by maintaining the integrity of cellular structures, scavenging ROS, and protecting the transcriptional and translational machinery of cyanobacteria (Hussain Wani et al., 2013). Additionally, proline inhibits the denaturation of enzymes, elevates the thermo-tolerance of enzymes, and acts as a buffer for cellular redox potential (Berard et al., 2015; Ngumbi and Kloepper, 2016).

### Dehydration effect on stress biomarkers

Stresses aggravate the generation of ROS viz- singlet oxygen hydroxyl radical, superoxide, and hydrogen peroxide and cause oxidative damage in cells (Sharma et al., 2013). The estimation of MDA content is used as a lipid peroxidation marker in studies related to oxidative stress. ROS may interact with macro- and micro-molecules, obstructing cellular processes. ROS are regarded as highly reactive molecules, even though they are considered crucial components of aerobic life due to their direct or indirect role in stress adaptation and the regulation of plant development from germination to senescence (Morales and Munné-Bosch, 2019). Different organelles, such as mitochondria and chloroplasts, serve as both the source and the initial target of ROS generated under drought stress (Farooq et al., 2009). ROS are highly reactive and quickly oxidize other target molecules, which leads to lipid peroxidation among other biochemical reactions. ROS production and lipid peroxidation are both fundamental aspects of aerobic life and essential traits of photosynthetic organisms. Therefore, both nonenzymatic and enzymatic lipid peroxidation processes may lead to the formation of MDA and other lipid peroxidation products in plants (Weber et al., 2004; Farmer and Mueller, 2013). MDA is a main constituent of lipid peroxidation, which is caused by free radicals oxidizing polyunsaturated fatty acids. In *N. calcicola* BOT1, MDA contents were significantly increased in AD and DD mats at 6 h of dehydration; they were (0.0663 ± 0.000 µ mol/mg DW) and (0.0659 ± 0.000 µ mol/mg DW, i.e., 1.09 and 1.09 fold higher than the control, respectively. Beyond increasing the duration of dehydration, decreased MDA content was found in the cells of DD mats, while there was no significant change in AD mats (**Figure 5C**). Our obtained result also correlates with the findings of Christou et al. (2013), who studied strawberries. In contrast, increasing the duration of dehydration increased the H_2_O_2_ content, and it reached its maximum at 24 h of dehydration treatment in both AD and DD samples, at (0.289 ± 0.029 µ mol/mg DW) and (0.2146 ± 0.003 µ mol/mg DW), i.e., 17 and 12.5 fold higher than the control (**Figure 5D**). H_2_O_2_ concentration decreased in further dehydration treatment compared to 24 h treatments. In the current study, high H_2_O_2_ buildup in *N. calcicola* BOT1 at 24 h of dehydration may have amplified the Haber-Weiss reaction, causing the generation of hydroxyl radicals, which in turn caused severe lipid peroxidation in cell organelles and plasma membrane destruction (Mittler, 2002). ROS generation may cause changes in microalgal metabolism like decreased CO_2_ fixation, nutrient uptake, photoreduction, and damage to the reaction center (Fal et al., 2022). In contrast, the production of H_2_O_2_ is essential for the synthesis of plant hormones like brassinosteroids, jasmonates, and abscisic acid, which improve plant, algae, and cyanobacterial stress tolerance (Zhou et al., 2014; Qu et al., 2021). The regulation of various physiological and biochemical processes related to plant growth and stress tolerance depends on signaling molecules, like jasmonates, and their derivatives (Kazan, 2015). Several cyanobacteria, including *Anabaena, Cylindrospermum, Calothrix, Nostoc, Spirulina*, *Synechococcus* sp., and *Scytonema* have been found to synthesize and produce jasmonate in previous studies (Ueda et al., 1991; Tsavkelova et al., 2006).

The depleted ROS level after 24 h of treatment may be due to the suppression of ROS production and the enhancement of anti-oxidative enzymes, total carotenoid, TPC, TFC, and proline, which inhibit the H_2_O_2_, and MDA content. This suggests that the *N. calcicola* BOT1 was under extreme stress to repair the damage brought on by dehydration stress (Bieker and Zentgraf, 2013). In our study, compatible solutes like carotenoid, proline, TPC, and TFC were enhanced under dehydration conditions from 6 to 96 h to reduce MDA and H_2_O_2_ accumulation. Additionally, the substantial negative connection between MDA, H_2_O_2_, pigments, and protein suggests that oxidative stress brought on by dehydration-induced MDA and H_2_O_2_ generation may be one of the factors inhibiting pigment production in *N. calcicola* BOT1.

### Dehydration effect on non-enzymatic assay

The TFC and TPC are potent ROS scavengers that maintain the integrity of the membrane and insulate cells against oxidative stress (Mukherjee et al., 2020; Fal et al., 2022). In this investigation, *N. calcicola* BOT1 showed a significant (p<0.05) rise in TFC and TPC in dehydrated mats (**Figures 5E, F**). The TFC in isolated *N. calcicola* BOT1 was at its maximum in 48 h dehydrated mats of AD and DD (**Figure 5E**), at (0.041 ± 0.001 μg QE/mg DW) and (0.045 ± 0.001 TFC μg QE/mg DW), i.e., 1.9 and 2.1-fold greater than the control. TPC was always greater in dehydrated mats compared to the control (**Figure 5F**). TPC was at its maximum in 6 h AD and DD mats, at (0.567 ± 0.009 μg GAE/mg DW) and (0.538 ± 0.009 TPC μg GAE/mg DW), which was 1.34 and 1.27-fold greater than the control. A previous study also found that *Chlamydomonas reinhardtii* and *Acutodesmus dimorphus* significantly increased their TFC and TPC levels under stress conditions (Chokshi et al., 2017). Reddy et al. (2004) also reported an ameliorated response of TFC and TPC under drought stress in higher plants.

### Vital staining by dual fluorescence dye

**Figure 7:**
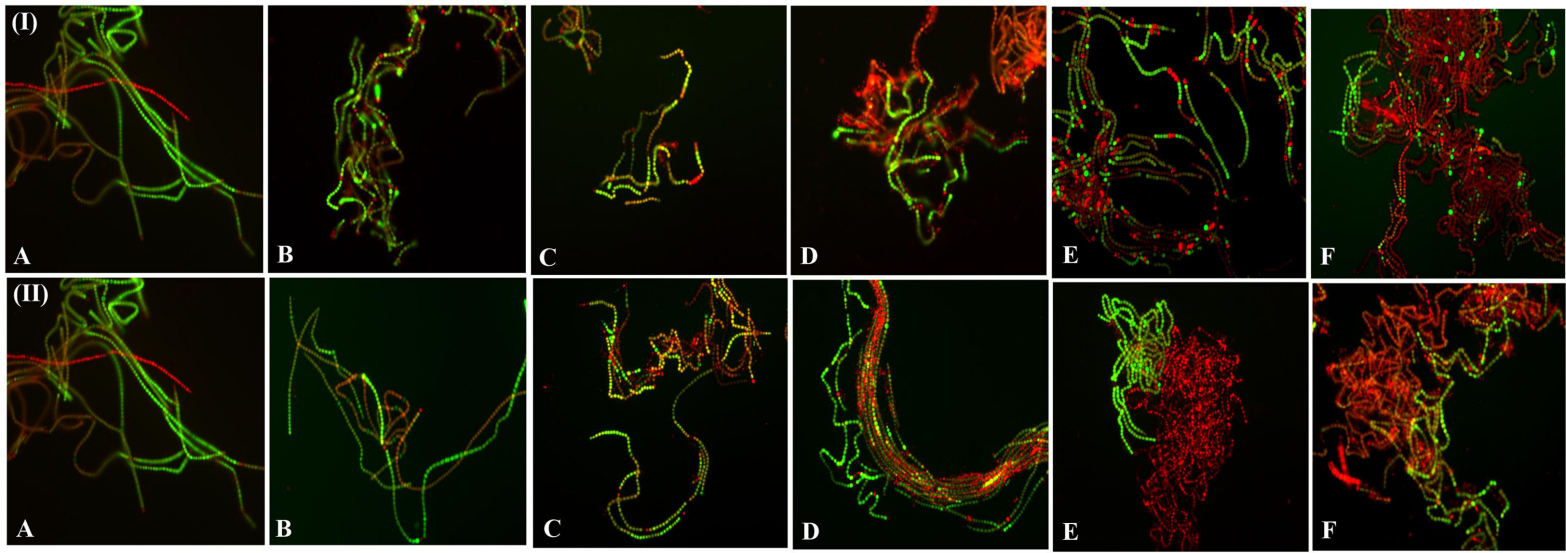
Fluorescence microscopic photographs (at 20X magnification) of *N. calcicola* BOT1 at different durations of dehydration treatment. Cyanobacterial mats were stained with FDA and PI. Live cells were stained with FDA and fluorescent green, while dead cells were stained with PI, fluorescent red, and yellow. (I) air-dried mats and (II) desiccator-dried mats in which **(A)** indicates control, and **(B–F)**, denote dehydration treatments for 6, 12, 24, 48, and 96 hours, respectively.

Fluorescence-based live-dead assays of the cells during dehydration treatments are presented in **Figure 7**. The green and red fluorescence intensity distributions in live-dead filaments were stained with FDA and PI. PI is a nuclear-binding dye; damaged or dead cells allow the entry of PI inside the cell, while a live cell does not (Deligeorgiev et al., 2009). In contrast, FDA is taken up by cells, which results in the conversion of non-fluorescent to fluorescent green metabolite fluorescein and stains the live or viable cell (Gumbo et al., 2014). Fluorescence microscopic images confirmed that dehydration acts in a dose-dependent manner, i.e., with a gradual increase in the duration of the dehydration treatment, dead cells increased (**Figure 7**). The results indicate that decreased water content increased the live-dead cell ratio. Fluorescence microscopy also showed morphological changes in the filaments and their compactness. Microphotographs showed a positive correlation with photosynthetic efficiency, pigment composition, and ROS production in cells. PI and FDA fluorescent staining were used by Yewalkar et al. (2019) for the determination of live-dead status under a photobioreactor for *Chlorella pyrenoidosa, Synechococcus* 7002, *Scenedesmus dimorphus*, and *Synechococcus elongatus* 7942. The dual-staining technique was also used by Fan et al. (2013) to detect the viability of *Microcystic* sp. after exposure to ultrasonic radiation.

### Metabolites profiling through UHPLC-HRMS

**Figure 8:**
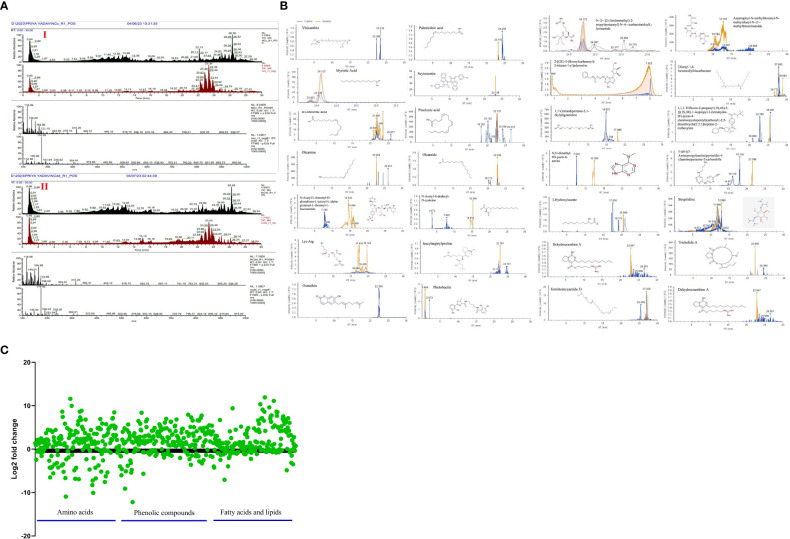
**(A)** Chromatogram obtained by UHPLC-HRMS analysis of the control and treated sample in positive and negative ion mode, **(B)** extracted ion chromatogram and chemical structure of some selected metabolites and graph, **(C)** down-regulated and up-regulated metabolites after dehydration treatment.

Utilizing UHPLC-HRMS, the metabolite profiles of *N. calcicola* BOT1 control and 96 h DD mats were examined. The base peak chromatograms of the control and 96 h DD mats were obtained *via* negative and positive ion mode, and the extracted ion chromatograms (EICs) for some of the significant identified compounds are shown in **Figures 8A**, **B**. Compounds were identified based on precise mass measurements, elemental composition, and analysis of the fragmentation pattern generated through MS2 sign. The names of the detected compounds, their chemical formulas, Annot. DeltaMass (ppm), computed molecular weights, m/z values, retention times, Log2 fold change (indicating up and down-regulated metabolites), and peak areas are listed in **Table 2** and **Supplementary Table 1**, respectively. According to Log2 fold change, the majority of the chemicals were elevated throughout the 96 h desiccator dehydrating mat treatment.

**Table 2:** List of major compounds identified by UHPLC-HRMS analysis through negative and positive ion mode, showing chemical formula, Annot.

Metabolites	Formula	Annot. DeltaMass (ppm)	Calc. MW	m/z	RT [min]	Log2 Fold Change: (Treated)/(Control)	Area (Max.)
Vitixanthin	C_33_H_42_O_6_	4.99	534.3008	533.2936	23.34	1.67	5730511.16
Palmitoleic acid	C_16_H_30_O_2_	0.36	254.2247	571.91	23.173	0.312	1912670.34
Myristic Acid	C_14_H_28_O_2_	0.36	228.209	182.81	24.205	1.023	13944229.86
Scytonemin	C_36_H_20_N_2_O_4_	0.24	544.1424	451.21	23.165	.07	30145757.4
α-Linolenic acid	C_18_H_30_O_2_	0.39	278.2247	416.29	22.465	0.173	4545133.92
Pinolenic acid	C_18_H_30_O_2_	-0.6	278.2244	461.52	22.667	.99	3517728.04
Oleamine	C_18_H_37_N	0.3	267.2927	142.87	22.661	.07	1742349.93
Oleamide	C_18_H_35_NO	0.23	281.2719	526.72	22.704	.21	5638042.91
N-Acetyl-L-threonyl-O-phosphono-L-tyrosyl-L-alpha-glutamyl-L-threonyl-L-leucinamide	C_30_H_47_N_6_O_14_P	1.22	746.2897	374.1521	15.49	8.64	28081785.5
N-Acetyl-S-dodecyl-D-cysteine	C_17_H_33_NO_3_S	-0.33	331.218	252.02	15.333	1.09	1261055.12
Lys-Arg	C_12_H_26_N_6_O_3_	1.47	302.2071	303.2141	20.151	1.69	12302196.3
leucylarginylproline	C_17_H_32_N_6_O_4_	-4.95	384.2466	383.2393	22.792	1.76	4200668.53
N~2~-[2-(Aminomethyl)-2- propylpentanoyl]-N~6~-carbamimidoyl-L-lysinamide	C_9_H_18_N_8_O	2.74	254.1611	255.1684	9.085	3.99	72178533.1
Asparaginyl-N-methylthreonyl-methylalanyl-N~2~- methylthreoninamide	C_18_H_34_N_6_O_7_	3.1	446.2503	445.2432	10.59	4.43	8267989.56
2-[(2E)-3-(Benzylcarbamoyl)-2-triazen-1-yl]adenosine	C_18_H_21_N_9_O_5_	0.19	443.1667	442.1594	7.914	4.31	137634103
Dioctyl 1,6-hexanediylbiscarbamate	C_24_H_48_N_2_O_4_	2	428.3623	429.3696	28.145	2.78	26242646.6
1,1’-(iminodipentane-5,1-diyl)diguanidine	C_12_H_29_N_7_	2.22	271.2491	272.2563	26.05	6.29	19082853.1
1,1,1-Trifluoro-2-propanyl (1S,4S)-5-{[(1S,3R)-1-isopropyl-3-(tetrahydro-2H-pyran-4-ylamino)cyclopentyl]carbonyl}-2,5-diazabicyclo[2.2.1]heptane-2-carboxylate	C_23_H_36_F_3_N_3_O_4_	1.26	475.2664	476.2737	25.501	5.86	13290558.3
N,N-dimethyl-9H-purin-6-amine	C_7_H_9_N_5_	0.54	163.0859	164.0932	12.4	7.43	49764270.2
5-((6-((3-Aminopropyl)amino)pyrimidin-4-yl)amino)pyrazine-2-carbonitrile	C_12_H_14_N_8_	2.43	270.1348	271.1421	21.43	5.4	11391203.4
3-Hydroxylaurate	C_12_H_23_O_3_	4.85	215.1658	216.173	20.492	4.06	28678283.4
Photobactin	C_22_H_25_N_3_O_7_	-3.61	443.16765	478.13786	0.866	2.13	22725890.45
Streptidine	C_8_H_18_N_6_O_4_	4.23	262.1401	261.1328	12.186	2.41	8162339.66
Dehydrocrambine A	C_24_H_42_N_6_O_2_	-2.15	446.336	447.343	22.841	3.56	4273424.71
Tricholide A	C_24_H_41_NO_4_	-4.82	407.3016	406.2938	22.064	3.53	721042.996
Ostruthin	C_19_H_22_O_3_	4.84	298.1583	297.1511	22.101	2.31	399576947
Termitomycamide D	C_24_H_44_N_2_O_3_	1.82	408.3359	409.3434	27.009	6.16	146232397

DeltaMass (ppm), cal. MW (calculated molecular weight), m/z value, RT (Retention Time), Log2 fold change, and peak area of control and 96 h DD mats in negative and positive ion mode.

Metabolite changes during dehydration included nucleotide derivatives, amino acids, carbohydrates, antioxidants, polyamines, lipids, and some defense compounds. The metabolomics analyses in the control and dehydrated mats revealed several significant compounds, which are listed below (**Table 2**; **Supplementary Table 1**). The 96-hour dehydrated mats metabolic profile was different from that of the control. The more prevalent metabolites were amino acids followed by peptides, nucleotides, lipids, and secondary metabolites.

While under dehydration stress, the amino acids, peptides, and nucleotide metabolites were overrepresented (Yobi et al., 2013). Several plants have been shown to accumulate more amino acids when exposed to drought stress (Bowne et al., 2012; Silvente et al., 2012; Hochberg et al., 2013; Rahman et al., 2017). Some unknown metabolites were more prevalent under dehydration stress than they were under hydrated conditions, indicating that they may play a part in desiccation tolerance. Dehydration causes the metabolism to shift in favor of producing carbohydrate, antioxidant, and nitrogen remobilization (Oliver et al., 2011). While fully hydrated mats contain large amounts of alanine and glycine (Khan et al., 2019), amino acids such as lysine, threonine, glutamine, aspartate, glutamate, arginine, asparagine, N~5~-(Diaminomethylene)-L-ornithylglycinamide, leucylarginylproline N-Acetyl-S-dodecyl-D-cysteine, Fluoro[bis(2-methyl-2-propanyl)]2-propyn-1-ylsilane, N-Acetyl-L-threonyl-O-phosphono-L-tyrosyl-L-alpha-glutamyl-L-threonyl-L-leucinamide, (E)-N~6~-(1-Aminoethylidene)-N-2H-tetrazol-5-yl-L-lysinamide are prominent in dehydrated mats. Martinelli et al. (2007) and Oliver et al. (2011) observed the accumulation of arginine, asparagine, glutamine, glutamate, and quinate during desiccation in *Sporobolus stapfianus*. According to Hoekstra et al. (2001), alterations in lipid compositions are crucial for desiccation tolerance because maintaining membrane integrity is crucial for dehydration tolerance. Under dehydration stress, a number of fatty acids, including oleic acid, palmitoleic acid, myristic acid, linolenic acid, pinolenic acid, oleamine, and oleamide, are overrepresented in order to preserve the integrity of the membrane. Periodic desiccation also promotes the synthesis of scytonemin in *Chroococcidiopsis* sp. (Fleming and Castenholz, 2007), which may be another protective mechanism for survival under dehydration stress. 3-Hydroxylaurate is a medium-chain fatty acid anion and a 3-hydroxy fatty acid anion. A polycyclic guanidine alkaloid called dehydrocrambine A prevents HIV-1 fusion (Chang et al., 2003). Ostruthin, Tricholide A, Termitomycamide D, and Streptidine show prominent antimicrobial, antibacterial, cytotoxic, and antimycobacterial activity (Schinkovitz et al., 2003; Choi et al., 2010; Bertin et al., 2017).

### Cyanobacteria’s mechanisms of adaptation to dehydration stress and their function in reducing drought stress on plants

**Figure 9:**
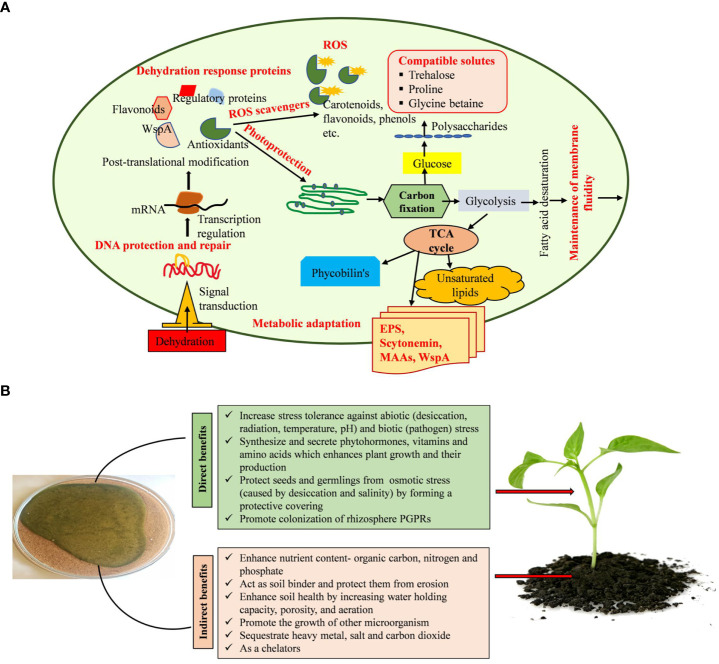
**(A, B)**. Mechanisms used by cyanobacteria in alleviating dehydration stress and plant growth promotion.

The variety of cyanobacterial species is a primary constituent of biocrust, which can withstand desiccation, extremely high or low temperatures, salinity, and pH (Singh and Jha, 2016). Acclimation strategies employed by cyanobacteria to survive under dehydration stress, including the synthesis and production of EPS, maintenance of ion channels, up-regulation of the DNA repair system, chaperone recruitment to maintain protein and enzyme integrity, synthesis of various compatible solutes, and oxidative stress protection system (**Figure 9A**), were used to adapt to low levels of available water (Nelson et al., 2021; Yadav et al., 2021; Yadav et al., 2022b). Compatible solutes like water stress proteins (WspA), sucrose, trehalose, glycine betaine, and proline inhibit protein denaturation and unfolding, stabilize macromolecules, and protect against osmotic imbalance under low water potential (Dabravolski and Isayenkov 2022; Potts, 1999). Several cyanobacterial genera discharge EPS into the environment, which enhances soil stability, water retention, and crusting (Rossi and De Philippis, 2015; Rossi et al., 2017). The EPS produced by cyanobacteria is composed of numerous components and serves a variety of functions throughout its life cycle, including symbiosis and predation protection. EPS also protects the cell from various types of stresses like ultraviolet radiation, temperature, desiccation, and salinity stress (Adhikary and Sahu, 1998; Potts, 2004; Van Hijum et al., 2006). EPS bears the features of chelating metal ions and buffering H_2_O_2_ (Hao et al., 2013; Gao et al., 2015; Zhang et al., 2013; Wang et al., 2014). WspA protein, glycan, mycosporine-like amino acids (MAAs), and scytonemin constitute the majority of the EPS matrix (Wright et al., 2005; Liu et al., 2017). EPS absorbs dew, vapor, and fog for the physiological repair of the cells (Agam and Berliner, 2006; Rao et al., 2009). In addition, serving as a barrier against desiccation, the presence of several functional groups, such as carboxyl, carbonyl, sulfate, and hydroxyl, in cyanobacteria’s EPS allows it to accumulate heavy metals (Pereira et al., 2011). It was discovered that the polysaccharides secreted by *Dunaliella salina* activate the jasmonic acid pathway, a metabolic system involved in plants’ response against stress, lowering the damages caused by salt stress in tomato cultures (El Arroussi et al., 2018). Similarly, seaweed extracts from *Fucus spiralis* and *Ulva rigida* were applied to bean plants to promote growth and increase their resistance to drought (Mansori et al., 2015).

EPS can absorb and hold water, generating a gelatinous coating around the cell and managing water intake and water loss due to its hydrophobic and hydrophilic properties (Adhikary and Sahu, 1998; Caiola et al., 1996). Additionally, it supplies organic carbon and nitrogen, solubilizes and mobilizes phosphorus, boosts water retention, and synthesizes and secretes certain phytohormones, all of which have the ability to promote the germination and growth of desert plants (**Figure 9B**) (Song et al., 2017). The resulting biocrust will have an impact on the emergence and establishment of new plants in arid and semi-arid places (Muñoz-Rojas et al., 2018; Parsons, 2012; Xu et al., 2013). The EPS released by cyanobacteria has a major role in the protection of cells from salinity and desiccation by developing an outer buffer zone (Pointing and Belnap, 2012; Rossi et al., 2017; Chittapun et al., 2018). Cyanobacteria enhance the general functionality of higher plants, promoting their growth, and encourage the formation of antioxidant chemicals, which increases the plants’ endurance in stress situations. The application of cyanobacteria as a biostimulant for crops under conditions of high or low temperatures, drought, and salt stress has proved successful (El Arroussi et al., 2018; Haggag et al., 2018). Cyanobacteria promote the growth of other bacteria, residing in the rhizosphere of plants (Badri et al., 2009; Noumavo et al., 2016). By defending plants from phytopathogens and synthesizing substances including hydrogen cyanide, antibiotics, and induced systemic resistance, these bacteria indirectly promote plant growth (Glick, 2014; Olanrewaju et al., 2017). It may be possible to promote plant development in challenging environmental conditions using phytohormones produced by cyanobacteria (Kaushal and Wani, 2016). These phytohormones help plants cope with abiotic challenges and increase their chances of survival (Fahad et al., 2015; Vurukonda et al., 2016). Many phytohormones, including cytokinins, auxins, gibberellins, jasmonates, abscisic acid, and ethylene, either stimulate shoot development or control growth-inhibitory plant processes, including senescence, abscission, and dormancy, regulating plant growth activities (Liu et al., 2012; Ahmed and Hasnain, 2014). Through all these activities, cyanobacteria promote the development and growth of plants in semi-arid and arid areas.

## Conclusion

The aquatic halotolerant cyanobacterium *N. calcicola* BOT1 was found to be tolerant to moderate desiccation but sensitive to extreme dehydration despite its formation of microscopic colonies with extracellular polysaccharides. In response to dehydration, *N. calcicola* BOT1 accumulated trehalose and proline. Although trehalose deposition is often associated with excessive desiccation tolerance, it has previously been thought to be significant to desiccation tolerance. Understanding the alterations that occur in dehydrated cells is necessary to comprehend the process of dehydration tolerance. It may be possible to determine the most plausible causes for why some cells are sensitive to desiccation while others are not through further research aimed at understanding the mechanism underlying desiccation tolerance. These studies also offer the intriguing potential for biocrust development and the biofertilizer potential of cyanobacteria in semi-arid and arid areas. We were able to recognize the metabolites in dehydrated mats and detect metabolic alterations brought on by dehydration stress attributable to an improved HRMS technique. The study also highlights the functions of lipids, fatty acids, amino acids, and phenolic substances in the biochemical and physiological adjustment of *N. calcicola* BOT1 to dehydration stress. The morphological, physiological, and biochemical changes that occur during acclimation raise the possibility that these particular biomolecules have a significant impact on desiccation tolerance.

## Data availability statement

The datasets presented in this study can be found in online repositories. The names of the repository/repositories and accession number(s) can be found in the article/**Supplementary Material**.

## Author contributions

PY and RKG: Designed study, Methodology, Writing Original Draft, Visualization; RPS: Validation, Investigation, Resources, Writing- Review, Data curation; HAA: Investigation, Writing- Original Draft, Review and editing; AAH: Investigation, Writing- Original Draft, Review and editing; GS: Investigation, Writing- Original Draft, Review and editing; AK: Investigation, Writing- Original Draft, Review and editing; RKG: Writing-Review and editing, Supervision. All authors contributed to the article and approved the submitted version.
