# Supplementary material for: Impact of dehydration on the physiochemical properties of Nostoc calcicola BOT1 and its untargeted metabolic profiling through UHPLC-HRMS
Source: Front Plant Sci. 2023 Jun 23;14:1147390. doi: 10.3389/fpls.2023.1147390 (PMC10327440; doi:10.3389/fpls.2023.1147390)
Supplement: Supplementary file 1 [file Table_1.doc]

**Supplementary Table 1** List of compounds identified by UHPLC-HRMS analysis through negative and positive ion mode, showing: chemical formula, Annot. DeltaMass (ppm), cal. MW (calculated molecular weight) m/z value, RT (Retention Time), Log2 fold change, and peak area of control and 96 h DD mats in negative and positive ion mode.

| **Metabolites** | **Formula** | **Annot. DeltaMass [ppm** | **Calc. MW** | **m/z** | **RT [min]** | **Log2 Fold Change: (Treated)/ (Control)** | **Area (Max.)** |
| --- | --- | --- | --- | --- | --- | --- | --- |
| 1,3-Dimethyl-8-(4-methyl-piperidin-1-ylmethyl)-7-(2-morpholin-4-yl-ethyl)-3,7-dihydro-purine-2,6-dione | C_20_H_32_N_6_O_3_ | -1.23 | 404.2531 | 422.2869 | 26.013 | 7.03 | 29298522.5 |
| Heneicosylic acid | C_21_H_42_O_2_ | -4.65 | 326.317 | 327.3234 | 26.329 | -0.5 | 174772.878 |
| L-Histidine | C_6_H_9_N_3_O_2_ | -4.15 | 155.0688 | 154.0615 | 19.615 | -0.73 | 3449176.14 |
| N~2~-[Amino(methoxyamino)methyl]-N~4~-ethyl-N~6~-isopropyl-1,3,5-triazine-2,4,6-triamine | C_10_H_22_N_8_O | 2.04 | 270.1922 | 271.1996 | 7.915 | -4.32 | 3373359.91 |
| 12α-Deoxy-deoxysaxitoxin | C_9_H_16_N_6_O | 3.94 | 224.1394 | 223.1322 | 18.499 | 1.3 | 5899105.99 |
| 1-(4-Nitrophenyl)-1H-pyrazolo[3,4-d]pyrimidin-4-amine | C_11_H_8_N_6_O_2_ | 3.86 | 256.0719 | 255.0646 | 16.735 | -8.28 | 28487625.9 |
| Hexahelicene | C_26_H_16_ | 3 | 328.1262 | 329.1335 | 16.943 | 3.55 | 4985286.38 |
| Fluoro[bis(2-methyl-2-propanyl)]2-propyn-1-ylsilane | C_11_H_21_FSi | -0.56 | 200.1395 | 199.1321 | 16.961 | 1.77 | 43281714.3 |
| beta-Hydroxypalmitate | C_16_H_31_O_3_ | 2.47 | 271.228 | 272.2353 | 17.292 | 4.61 | 3305141.46 |
| Sulcatol | C_8_H_16_O | -4.85 | 128.1195 | 129.1266 | 17.674 | -5.36 | 4742858.4 |
| N~5~-(Diaminomethylene)-L-ornithylglycinamide | C_8_H_18_N_6_O_2_ | 4.04 | 230.1501 | 229.1428 | 17.924 | 2.13 | 14239067.1 |
| N,N',N''-Trimethyl-1,4,7-triazonane-1,4,7-tricarboxamide | C_12_H_24_N_6_O_3_ | 1.68 | 300.1915 | 299.1846 | 18.022 | 5.43 | 99051603.3 |
| Amdoxovir | C_9_H_12_N_6_O_3_ | 3.76 | 252.098 | 251.0908 | 18.194 | -0.05 | 161963234 |
| oxonazine | C_9_H_14_N_6_O | 3.91 | 222.1238 | 221.1165 | 18.229 | -0.2 | 48415959.9 |
| N,N'-Dibutylsulfuric diamide | C_8_H_20_N_2_O_2_S | -3.61 | 208.1238 | 209.131 | 18.291 | -1.51 | 2238180.35 |
| 4-Amino-1-[3-({3-[(3-aminopropyl)amino]propyl}amino)propyl]-2(1H)-pyrimidinone | C_13_H_26_N_6_O | 2.63 | 282.2176 | 281.2106 | 23.254 | 3.02 | 4575995.64 |
| (3S)-5-{[(Diethylamino)methyl]amino}-3-({2-[3-{[(4-methyl-1,2,5-oxadiazol-3-yl)methyl]amino}-2-oxo-1(2H)-pyrazinyl]butanoyl}amino)-4-oxopentanoic acid | C_22_H_34_N_8_O_6_ | 3.45 | 506.2619 | 505.2546 | 23.273 | 0.99 | 13410897.9 |
| 2-amino-9-({[(2S)-1-azido-3-hydroxypropan-2-yl]oxy}methyl)-9H-purin-6-ol | C_9_H_12_N_8_O_3_ | 3.52 | 280.1042 | 279.0969 | 1.198 | 1.55 | 6.75E+08 |
| 2-{[5-(1,2-Dithiolan-3-yl)pentanoyl]amino}ethanesulfonic acid | C_10_H_19_NO_4_S_3_ | -1.57 | 313.0471 | 314.0544 | 3.487 | 0.23 | 2.25E+08 |
| N-(2-Methylbutyl)-N'-(6-{4-[5-(trifluoromethyl)pyridin-2-yl]piperazino}pyridin-3-yl)thiourea | C_21_H_27_F_3_N_6_S | 4.4 | 452.199 | 453.2063 | 3.506 | 0.37 | 3.13E+09 |
| L-Homocitrulline | C_7_H_15_N_3_O_3_ | -4.68 | 189.1105 | 420.2558 | 3.601 | 0.75 | 31496982 |
| Benzaldehyde | C_7_H_6_O | -3.17 | 106.0415 | 107.0485 | 3.693 | 3.68 | 42863195 |
| 3,5-Dimethyl-4-({5-[(4-nitrophenyl)amino]pyrazolo[1,5-a]pyrimidin-7-yl}oxy)benzonitrile | C_21_H_16_N_6_O_3_ | -0.27 | 400.1283 | 401.1356 | 13.402 | -6.65 | 99298365.1 |
| 1-hydrazinylhexose | C_6_H_14_N_2_O_6_ | -1.27 | 210.0849 | 209.0776 | 13.602 | 0.56 | 46410404.2 |
| 1-[(2S)-2,3-Dihydroxypropyl]-3-(3-methylphenyl)-1-(3,3,3-trifluoropropyl)urea | C_14_H_19_F_3_N_2_O_3_ | 1.67 | 320.1353 | 319.128 | 13.913 | -0.47 | 40982242.7 |
| 2-[Cyano-(4-dimethylamino-6-methoxy-[1,3,5]triazin-2-yl)-amino]-N,N-diethyl-acetamide | C_13_H_21_N_7_O_2_ | 2.88 | 307.1766 | 306.1693 | 14.559 | 3.21 | 19219447.3 |
| N-[4-(3-Amino-1,2-benzoxazol-4-yl)phenyl]-N'-(3-fluorophenyl)-1,1-cyclopropanedicarboxamide | C_24_H_19_FN_4_O_3_ | -3.63 | 430.1426 | 429.1353 | 14.725 | 0.14 | 3173951.11 |
| (E)-N-[(2-Carbamoylhydrazino)(hydrazino)methylene]glycine | C_4_H_10_N_6_O_3_ | 4.37 | 190.0823 | 189.075 | 14.73 | 0.42 | 2120128.48 |
| 6-(4-Methyl-1-piperazinyl)-2,4-pyrimidinediamine | C_9_H_16_N_6_ | 3.95 | 208.1445 | 207.1372 | 14.786 | 1.52 | 7452914.41 |
| Dibenzyl [(E)-1,2-ethenediylbis{4,1-phenyleneimino[(2S)-1-oxo-1,2-propanediyl]}]biscarbamate | C_36_H_36_N_4_O_6_ | -0.15 | 620.2634 | 638.2973 | 14.941 | -6.82 | 83404418.6 |
| Dimethylenetriurea | C_5_H_12_N_6_O_3_ | 3.91 | 204.09789 | 203.09061 | 1.098 | 2.48 | 17527542.72 |
| Terpineol | C_10_H_18_O | -0.34 | 154.1357 | 268.1 | 18.074 | 0.03 | 1483362.1 |
| Pyrenulic acid A | C_26_H_36_O_3_ | 2.55 | 396.2675 | 395.2602 | 25.358 | 0.18 | 16166864.6 |
| 1,1'-nonane-1,9-diyldiguanidine | C_11_H_26_N_6_ | 4.19 | 242.2229 | 241.2156 | 25.261 | -0.83 | 2933986.73 |
| N-[3-Fluoro-4-({6-methoxy-7-[3-(1-piperidinyl)propoxy]-4-quinolinyl}oxy)phenyl]-4-(4-fluorophenyl)-2-pyridinecarboxamide | C_36_H_34_F_2_N_4_O_4_ | 0.48 | 624.2551 | 623.2479 | 25.276 | 0.32 | 10319024 |
| Crambescidin 800 | C_45_H_80_N_6_O_6_ | 1.93 | 800.6155 | 799.6082 | 24.994 | 0.49 | 1674147.36 |
| 3-{4-[2-(4-Fluorophenyl)-2-(4-phenyl-1-piperazinyl)ethyl]-1-piperazinyl}-2-methyl-1-phenyl-1-propanone | C_32_H_39_FN_4_O | -2.11 | 514.3097 | 515.3168 | 25.113 | 4.86 | 5114592.85 |
| N-Cyclopentyl-N~2~-[2-ethyl-2-(isobutyrylamino)butanoyl]-L-argininamide | C_21_H_40_N_6_O_3_ | 0.92 | 424.3166 | 423.3093 | 24.878 | 0.31 | 3982200.67 |
| N~5~-(Diaminomethylene)-L-ornithyl-L-lysyl-L-lysine | C_18_H_38_N_8_O_4_ | 0.56 | 430.3018 | 429.2946 | 24.929 | -0.63 | 1109551.98 |
| (2E)-N-{4-[(3-Aminopropyl)amino]butyl}-N'-{6-[(diaminomethylene)amino]hexyl}-2-butenediamide | C_18_H_37_N_7_O_2_ | 1.2 | 383.3013 | 382.2941 | 24.958 | 1.22 | 4604028.84 |
| N-{1-[4-(2-Methoxyphenyl)-1-piperazinyl]-1-(2-thienyl)-2-propanyl}-2-thiophenecarboxamide | C_23_H_27_N_3_O_2_S_2_ | 2.62 | 441.1556 | 440.1484 | 24.976 | -0.06 | 2329408.53 |
| H-Phe-Arg-Arg-OH | C_21_H_35_N_9_O_4_ | 3.39 | 477.2828 | 476.2755 | 24.205 | -0.42 | 12875332.5 |
| Quinocinnolinomycin A | C_23_H_33_N_3_O_3_ | 2.95 | 399.2534 | 400.2607 | 12.243 | -3.76 | 25653593.9 |
| arg-gln | C_11_H_22_N_6_O_4_ | 2.94 | 302.1711 | 301.1639 | 19.245 | -0.15 | 8942006.48 |
| Biotin-azide | C_13_H_22_N_6_O_2_S | 2.37 | 326.1533 | 325.146 | 19.044 | -0.64 | 72880690 |
| 1-(Diethylsulfamoyl)-N-(4-fluorobenzyl)-4-piperidinecarboxamide | C_17_H_26_FN_3_O_3_S | 1.94 | 371.1686 | 370.1613 | 11.182 | -1.37 | 2928461.25 |
| 1-{4-[2-(2,2,2-Trifluoroethoxy)ethyl]-1-piperazinyl}-2-propanol | C_11_H_21_F_3_N_2_O_2_ | 3.3 | 270.1564 | 269.1491 | 11.672 | 2.52 | 55338147.2 |
| Methyl 3-({[4-(4-fluorophenyl)-1-piperazinyl]acetyl}amino)-5-methyl-1H-indole-2-carboxylate | C_23_H_25_FN_4_O_3_ | 2.56 | 424.1922 | 423.1849 | 11.878 | 3.03 | 2079418.69 |
| 1,1,1-Trifluoro-2-dodecanone | C_12_H_21_F_3_O | 2.94 | 238.1552 | 237.1479 | 19.862 | 1.7 | 28431380.3 |
| N,N'-Dicarbamimidoyldecanediamide | C_12_H_24_N_6_O_2_ | 2.5 | 284.1968 | 283.1897 | 19.613 | 4.8 | 39697457.9 |
| 1-(Cyanomethyl)-1-(2-pyridinylmethyl)pyrrolidinium | C_12_H_16_N_3_ | -0.4 | 202.1343 | 203.1416 | 20.194 | -0.25 | 467310473 |
| Siguazodan | C_14_H_16_N_6_O | 3.69 | 284.1396 | 283.1323 | 20.395 | 1.23 | 2406347.6 |
| Difenoxuron | C_16_H_18_N_2_O_3_ | -0.54 | 286.1316 | 285.1243 | 20.422 | -0.23 | 73771718 |
| N-Methylglycyl-L-alanyl-N~5~-(diaminomethylene)-L-ornithine | C_12_H_24_N_6_O_4_ | 3.36 | 316.187 | 315.1797 | 21.256 | -0.34 | 2381352.86 |
| N,N-Dibutylacetamide | C_10_H_21_NO | -3.8 | 171.1617 | 365.3135 | 21.289 | 1.19 | 5627990.83 |
| N,N'-Bis(4,6-dimethylpyrimidin-2-yl)piperazine | C_16_H_22_N_6_ | 3.03 | 298.1915 | 297.1842 | 21.184 | 0.84 | 1955608.14 |
| N-(Sulfanylacetyl)-L-tyrosyl-L-argininamide | C_17_H_26_N_6_O_4_S | -5 | 410.1716 | 409.1643 | 20.732 | -1.2 | 4487527.53 |
| N-[5-(4-Ethyl-1-piperazinyl)pentyl]-6,7-dimethoxy-2-(1-pyrrolidinyl)-4-quinazolinamine | C_25_H_40_N_6_O_2_ | -1.73 | 456.3205 | 457.3278 | 25.465 | 1.77 | 30788490.4 |
